# Spatiotemporally synchronized pure high-even-order dispersion solitons in multimode fiber lasers

**DOI:** 10.1038/s41467-026-78194-5

**Published:** 2026-10-03

**Authors:** Wenqi Ma, Xiuquan Li, Canyi Deng, Caishan Pang, Rui Nie, Frank W. Wise, Guijun Hu

**Affiliations:** 1 College of Communication Engineering, Jilin University, Changchun, China; 2 School of Applied and Engineering Physics, Cornell University, Ithaca, NY USA; 3 Jilin Provincial Key Laboratory of Specialty Optical Fiber Fabrication and Application, Changchun, China

**Keywords:** Fibre lasers, Ultrafast photonics

## Abstract

Soliton solutions of nonlinear wave equations continue to attract broad scientific interest across diverse physical settings. Currently, the study of pure high-even-order dispersion (PHEOD) solitons and multimode spatiotemporal dynamics represents two compelling frontiers in ultrafast photonics. The intersection of these domains is theoretically rich; however, the realization of PHEOD solitons in multimode fibers has been difficult due to the challenge of independently managing dispersion for different transverse modes. Here, we report the first experimental generation of synchronized PHEOD solitons in a multimode fiber laser. By employing a transverse mode division control technique, we physically decouple the modes, which enables control of both modal and chromatic dispersions. We systematically demonstrate the generation of spatiotemporally synchronized PHEOD solitons in four transverse modes, with fourth-, sixth-, or eighth-order dispersion. These multimode pulsed states self-organize through nonlinear coupling among the transverse modes. Numerical simulations account for the main experimental observations and illuminate the nonlinear multimode dynamics that underlie the laser operation. This work bridges the scientific gap between PHEOD engineering and multimode fiber lasers, and opens new possibilities for the development of sources of multidimensionally structured ultrafast light.

## Abstract (web summary)

Soliton solutions of nonlinear wave equations in diverse physical systems continue to attract scientific interest. Here, authors demonstrate spatiotemporally synchronized pure high-even-order dispersion solitons in a multimode fiber laser through dispersion engineering and transverse mode control.

## Introduction

Mode-locked lasers are crucial for ultrafast photonics and precise optical systems, with widespread applications in nonlinear microscopy^1,2^, micro- and nanofabrication^3^, ultrafast and high-resolution spectroscopy^4,5^, terahertz technology^6^, and all-optical storage^7,8^. Most modern femtosecond lasers rely on soliton-like pulse formation; pulses balance anomalous group-velocity dispersion (GVD, specifically second-order dispersion *β*_2_) and the Kerr nonlinearity (self-phase modulation, SPM)^9^. Although these solitons have been extensively studied, they are strictly limited by the soliton area theorem^10,11^: the pulse energy is inversely proportional to pulse duration, which significantly restricts the generation of high-peak-power ultrashort pulses in fiber lasers.

More broadly, soliton solutions of nonlinear wave equations in diverse settings continue to attract scientific interest, partly owing to their particle-like nature and partly because a wide variety of nonlinear phenomena can be understood in terms of soliton dynamics. A current frontier in the field of nonlinear waves is the study of pure high-even-order dispersion (PHEOD) solitons, in which SPM balances anomalous even-order dispersion. Blanco-Redondo et al. first demonstrated in dispersion-engineered photonic crystal waveguides^12^ that, when *β*_2_ is positive (normal), the balance between negative fourth-order dispersion (*β*_4_) and nonlinearity supports the pure-quartic soliton (PQS). Unlike conventional solitons, PQSs follow a scaling law with energy inversely proportional to the cube of the pulse duration^13^. Building on the work of Runge et al. regarding an infinite hierarchy of solitons^14^, this concept was subsequently extended to higher even-order dispersion mechanisms. It has been established that the balance of SPM with negative even-order dispersion of arbitrary order underlies PHEOD solitons. Pure sixth- (*β*_6_), eighth- (*β*_8_), and even tenth-order (*β*_10_) solitons^14,15^ have been observed experimentally. Theory and experiment reveal that as the dispersion order *k* increases, PHEOD solitons satisfy the energy-duration scaling law ${{{\rm{E}}}}\propto {\tau }^{-(k-1)}$, which is steeper than that of ordinary solitons. Recently, a mode-locked laser based on PQS formation was demonstrated^16^. This provides a flexible and reproducible testbed for the investigation of PHEOD solitons, and in principle, PHEOD solitons may offer advantages for the scaling of pulse energy in lasers with anomalous dispersion.

Multimode (MM) nonlinear phenomena have also attracted significant and growing attention in the past decade. Primary examples are MM soliton formation and MM mode-locking in lasers. A prototype MM soliton consists of phase-locked pulses in multiple transverse modes (TMs) of an optical fiber with anomalous dispersion^17^. Nonlinear interactions balance both the chromatic dispersion and the modal dispersion to form an MM soliton. The phase-locking of transverse and longitudinal modes of a laser resonator is referred to as spatiotemporal mode-locking (STML). The first STML lasers were based on dissipative-soliton formation in normal-dispersion cavities^18^, but there is also interest in STML in the anomalous-dispersion regime, where MM solitons can form^19,20^. In either regime, linear and nonlinear processes that counter chromatic and modal dispersion are critical to pulse formation. By achieving temporal synchronization and coherent superposition of multiple TMs, STML not only harvests energy from multiple modes but also mitigates excessive nonlinear phase accumulation via a significantly larger effective mode area. Thus, it offers significant potential for the generation of stable ultrashort pulses with high energy^21–25^.

MM solitons in systems with PHEOD are expected to exhibit a rich set of properties and dynamical phenomena, and this has motivated very recent theoretical treatments of MM PHEOD solitons in passive fiber^26^ and in lasers^27,28^. The experimental demonstration of such solitons faces major challenges: extreme values and precise control of both chromatic and modal dispersion are required. Current dispersion management techniques primarily rely on spectral pulse shapers^15,16^ or tailored microstructured waveguides^12,13^ to impress a dispersive phase on the optical field. In MM waveguides, the spatial overlap of different TMs makes it impossible to independently manipulate them using these methods, directly resulting in the inability to implement mode-specific control of higher-order dispersion. Consequently, PHEOD solitons have been confined to single-mode (SM) systems^13–16,29–31^. The experimental realization of MM mode-locking based on PHEOD soliton formation remains an open challenge. Experimental investigations of PHEOD soliton formation in MM fiber lasers will be scientifically fruitful, and it may be possible to scale the stable pulse energy by leveraging the combined properties of MM and PHEOD.

Here, we report the first experimental generation of synchronized PHEOD solitons in an MM fiber laser. This demonstration is facilitated by a transverse mode division control (TMDC) technique developed recently^25^. A TMDC module in the laser implements both i) a modal dispersion map and ii) control of the chromatic dispersion in the TM channels. PHEOD solitons governed by pure *β*_4_, *β*_6_, and *β*_8_ in each of four separate TMs are first generated and characterized systematically; these SM solitons exhibit ideal theoretical properties despite propagating in MM fibers. In MM operation, synchronized trains of solitons in individual modes self-organize due to intermodal nonlinear interactions in the laser. Numerical simulations agree with the experimental results and provide insight into the behavior and properties of the laser. Pulses in individual modes overlap temporally only in a portion of the cavity, so these new MM pulsed lasing states do not represent conventional STML. However, the laser exhibits some of the signatures of STML, and the relationship of the spatiotemporal wave propagation in the laser to STML will be discussed. Beyond the physics of nonlinear waves at the intersection of three conceptual frontiers—PHEOD solitons, MM solitons, and STML—as elucidated here, the experimental platform will underpin future sources of multidimensionally structured ultrafast light.

## Results

### Laser cavity

**Fig. 1:**
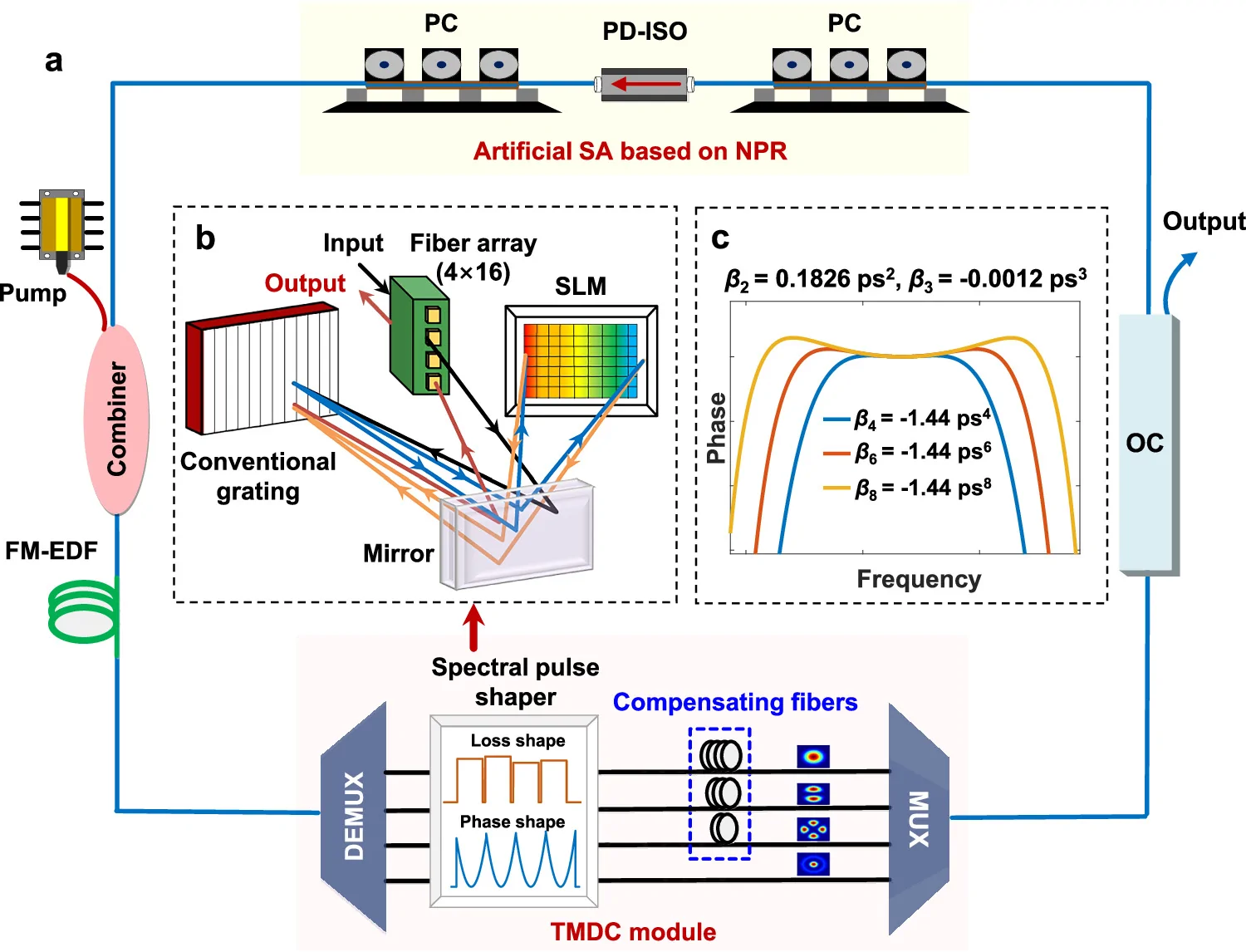
Experimental setup and dispersion management principle of the spatiotemporally synchronized PHEOD soliton laser. **a** Schematic of the laser cavity based on the transverse mode division control (TMDC) technique. The core TMDC module consists of a mode multiplexer/demultiplexer (MUX/DEMUX), a multi-channel spectral pulse shaper, and compensating fibers for balancing intermodal group delay (The mode profiles serve as logical identifiers for the corresponding intracavity modes, rather than the actual physical spatial distributions in the single-mode channels). FM-EDF, few-mode erbium-doped fiber; OC, output coupler; PC, polarization controller; PD-ISO, polarization-dependent isolator. **b** Internal optical architecture of the programmable spectral pulse shaper. Input beams are coupled via a fiber array and projected onto the spatial light modulator (SLM) through a diffraction grating and an imaging lens for phase modulation. **c** Target spectral phase profiles applied to the transverse mode channels. These profiles compensate for residual lower-order cavity dispersion, while simultaneously superimposing the target high-even-order dispersion.

The experimental setup is shown in Fig. 1a, and details are in the Methods section. An erbium-doped fiber (EDF) capable of supporting four TMs serves as the gain medium in the unidirectional ring cavity. Unless stated otherwise, the passive fiber in the cavity supports the same four modes. Nonlinear polarization rotation creates self-amplitude modulation of high-intensity pulses; it acts as an effective saturable absorber (SA), which is important for starting and stabilizing mode-locked states. When the laser is operated in multiple TMs, the self-amplitude modulation is spatiotemporal. A key component of the cavity is the TMDC module. To address the limitations caused by intermodal crosstalk in precise dispersion management, we use a customized cascade structure mode-selective coupler as the mode multiplexer/demultiplexer (MUX/DEMUX)^32^ to physically separate the components of the intracavity optical field. The MM beam is first spatially decomposed into independent TM channels via the DEMUX; subsequently, the isolated modes are coupled into different input ports of a spatial light modulator (SLM)-based programmable spectral pulse shaper (Finisar WaveShaper 16000 s, with the internal architecture shown in Fig. 1b). The separated channel pigtails of the mode MUX/DEMUX are SMF-28e fiber, while the port connected to the laser cavity is few-mode fiber (FMF). This architecture physically decouples the TMs, allowing the spectral pulse shaper to imprint higher-order dispersion profiles on each one. Furthermore, by utilizing compensating fibers to independently adjust the group delay for each spatial channel, the TMDC module can compensate for modal dispersion^33^. The TMDC module is positioned right before the SA based on numerical simulations that exhibit strong saturation of the absorber, which is expected to facilitate synchronization of solitons in distinct modes (Supplementary Fig. 3a). The total cavity length is about 8.3 m, corresponding to a fundamental repetition rate of 24.95 MHz. Additional details about the experimental setup are provided in the Methods section.

### Pure high-even-order dispersion in individual TMs

To generate PHEOD solitons, we use a two-step process with the pulse shaper. First, for each spatially resolved mode, we correct for the dispersion of the fibers and intracavity components. Due to pulse-shaper constraints, we adopted a maximum-dispersion-based strategy (see Supplementary Note 1), applying the same phase profile to each TM channel to imprint strong, negative high-even-order dispersion (up to *β*_8_) while effectively mitigating the impact of low-order dispersion (*β*_2_, *β*_3_). In our cavity, we adjust the net *β*_2_ of each TM to a small positive value^12^, while simultaneously ensuring that *β*_3_ is suppressed to negligible levels. This strategy establishes a robust physical environment that is conducive to the formation of PHEOD solitons.

Building on this baseline dispersion, we introduce strong, negative high-even-order dispersion. As shown by the phase profiles in Fig. 1c, we apply significant negative *β*_4_ (blue curve), *β*_6_ (red curve), or *β*_8_ (yellow curve) dispersion, respectively. The dispersion applied with the WaveShaper exceeds the higher-order dispersion of the fiber in the laser by several orders of magnitude. This ensures that soliton formation is due to the interaction of the specific high-even-order dispersion term and the Kerr nonlinearity. Although this approach introduces minor dispersion mismatches among the different TMs, quantitative comparisons of the spectral-phase and group-delay contributions, together with a sensitivity analysis, show that the residual terms do not compromise PHEOD soliton formation (see Supplementary Note 4). In experiments, all participating TMs entered the target dispersion regions, as confirmed by the soliton properties described below.

### Mode-locking with PHEOD soliton formation in individual TMs

We first investigate the characteristics of PHEOD solitons in individual TMs. To selectively excite specific TMs, we close the cavity using only the MUX/DEMUX channel corresponding to the target TM; all other channels are simply left unconnected. By increasing the pump power and adjusting the PCs, we observe that above a threshold of 1.1 W, a mode-locked pulse train at the cavity frequency is generated for the LP01 mode. The threshold increases for higher-order modes: 1.19 W for LP11, 1.35 W for LP02, and 1.56 W for LP21. The process is similar for each order of dispersion.

**Fig. 2:**
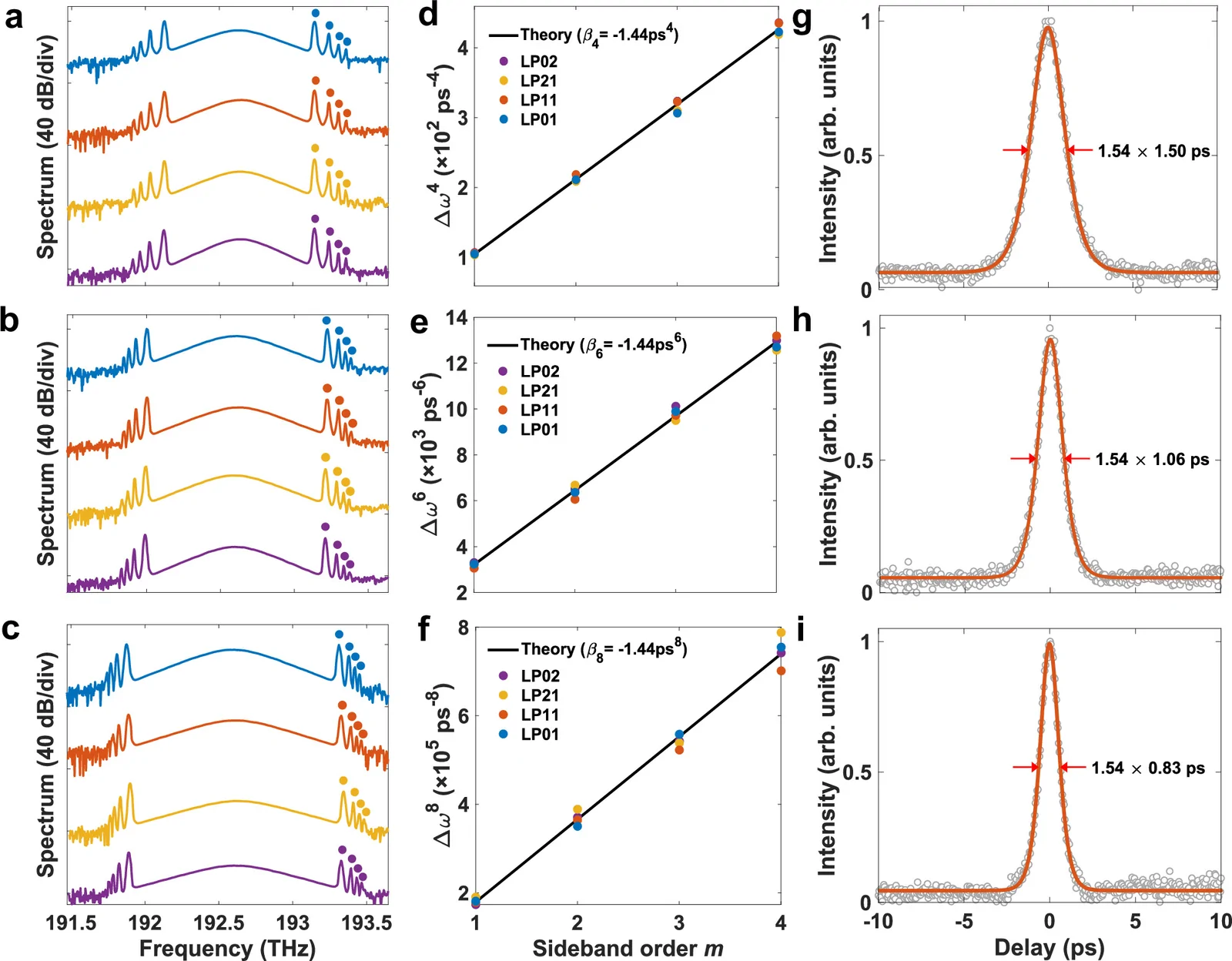
Spectral characterization of PHEOD solitons in individual TMs. Left column: Normalized experimental spectra recorded for four distinct TMs governed by pure *β*_4_ (**a**), *β*_6_ (**b**), and *β*_8_ (**c**). The dominant dispersion coefficients are set to *β*_k_ = −1.44 ps^*k*^. **d–f** Corresponding analysis of Kelly sideband positions, illustrating the linear dependence of the *k*-th power of the sideband frequency shift (Δ*ω*^*k*^) on the sideband index (*m*). Colored symbols denote distinct modes, while solid black lines indicate theoretical predictions. **g–i** Autocorrelation traces (ACs) of the LP11 mode at a dispersion of −1.44 ps^*k*^, along with their fits (red lines). The width differences for the other modes are within ±0.02 ps.

Perhaps the clearest experimental signature of PHEOD soliton formation in a laser is the appearance of strong resonant sidebands in the spectra, which arise from the same process as Kelly sidebands in ordinary soliton lasers^34^. As shown in the left column of Fig. 2, the mode-locked spectra of all excited TMs display these. As shown in the right column of Fig. 2, under regimes dominated by pure *β*_4_, *β*_6_, and *β*_8_, the *k*-th power of the sideband frequency shift exhibits a linear relationship with the sideband index *m* across all four TMs. Notably, however, for *β*_8_, we observe some deviations from the theoretical frequencies. While the variation is quite linear for the fundamental mode, higher-order modes (particularly LP21) start to deviate from the linear trend, especially for higher-order sidebands. Under our maximum-dispersion-based strategy, the intrinsic dispersion differences between TMs leave a small amount of uncompensated residual *β*_2_ and *β*_3_. As noted by Runge et al.^16^, the small discrepancies between the measured and theoretical positions of the sidebands could be due to this residual dispersion.

**Fig. 3:**
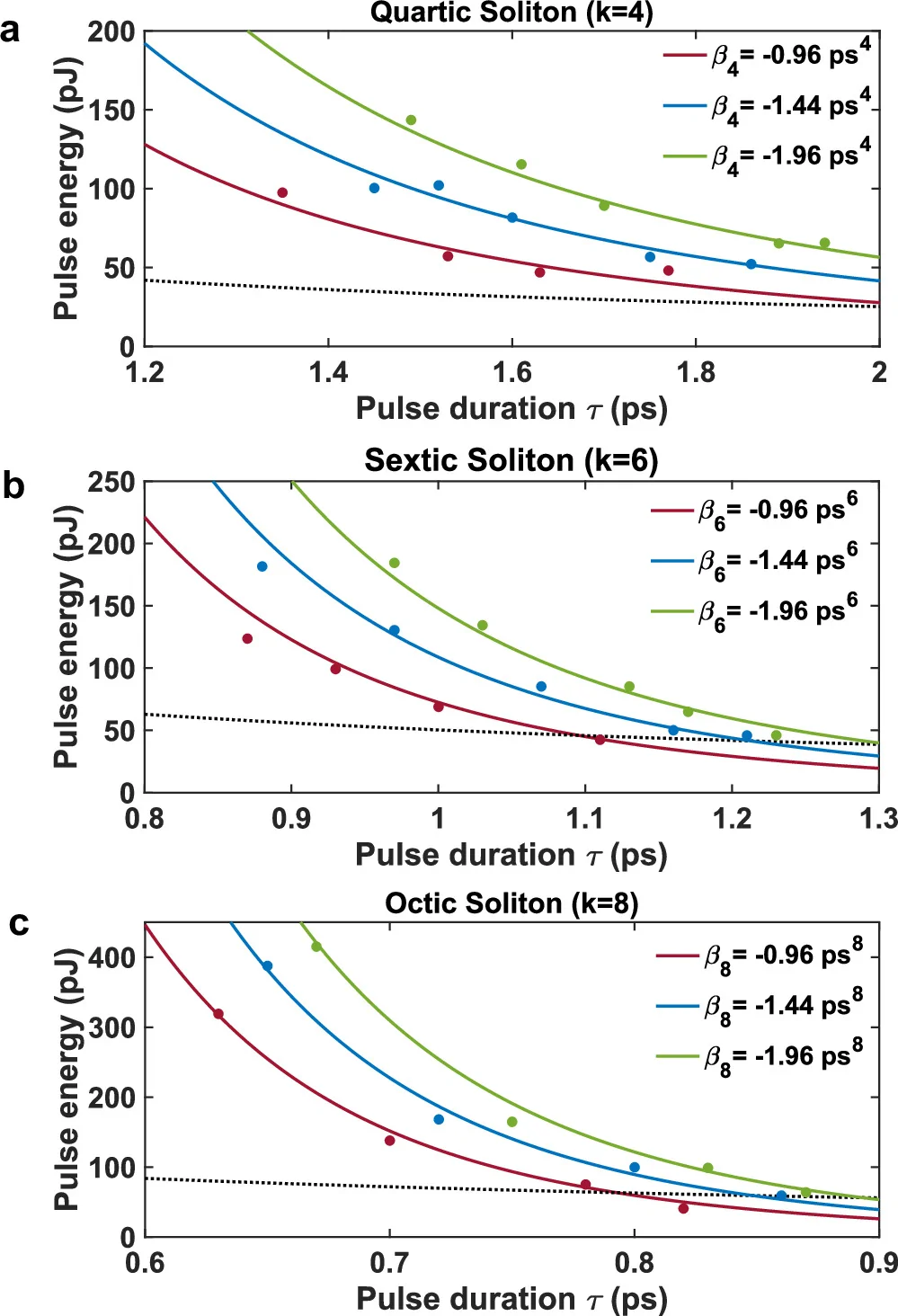
Energy-duration scaling of SM PHEOD solitons. **a**–**c** Experimental results and theoretical fits under dominant pure fourth- (**a**), sixth- (**b**), and eighth-order (**c**) dispersion. Distinct colors denote different net intracavity dispersion coefficients: red (−0.96 ps^*k*^), blue (−1.44 ps^*k*^), and green (−1.96 ps^*k*^). Solid circles and solid lines represent measured data and theoretical fits for SM PHEOD solitons. The black dashed lines denote the theoretical energy scaling of the SM *β*_2_ soliton (*β*_2_ = −0.1773 ps^2^, corresponding to the intrinsic net cavity dispersion without a phase mask).

The pulse energies increase with the order of dispersion, and overall they range from ~50 to ~400 pJ. Figure 3 shows the measured energy-duration relation for SM PHEOD solitons in the different higher-order dispersion regimes. The theoretical scaling of a conventional SM *β*_2_ soliton is also plotted as a comparative baseline. To enable a fair comparison, the SM data are collected within the same laser cavity. Using the TMDC module, we selectively block the channels corresponding to the higher-order modes (LP11, LP21, and LP02) at the spectral pulse shaper, thereby keeping the laser in a fundamental-mode (LP01) locked state. In this setup, we vary the pump power and measure the output pulse energy, using spectral integration to subtract the energy contained in the sidebands (see Supplementary Note 6). The data points in Fig. 3 correspond to a range of single-pulse energies for which mode-locked operation is stable and for which the intracavity pulse energy is below the WaveShaper damage threshold of 3 nJ. The data exhibit excellent agreement with the theoretical scaling (shown by solid lines in the figure).

All of the measurements of laser operation in a single TM agree with theoretical expectations and prior experiments. Thus, they demonstrate the generation of PHEOD solitons in the individual single modes. Despite the different mode-field distributions of the various TMs, the variation of the spectra with dispersion order is quite consistent, even with small imperfections in dispersion control. The systematic trends in the spectral shapes and sideband positions demonstrate the effectiveness of the TMDC technique for managing dispersion (see Supplementary Note 5 for results under additional dispersion regimes).

### Generation of spatiotemporally synchronized PHEOD solitons

**Fig. 4:**
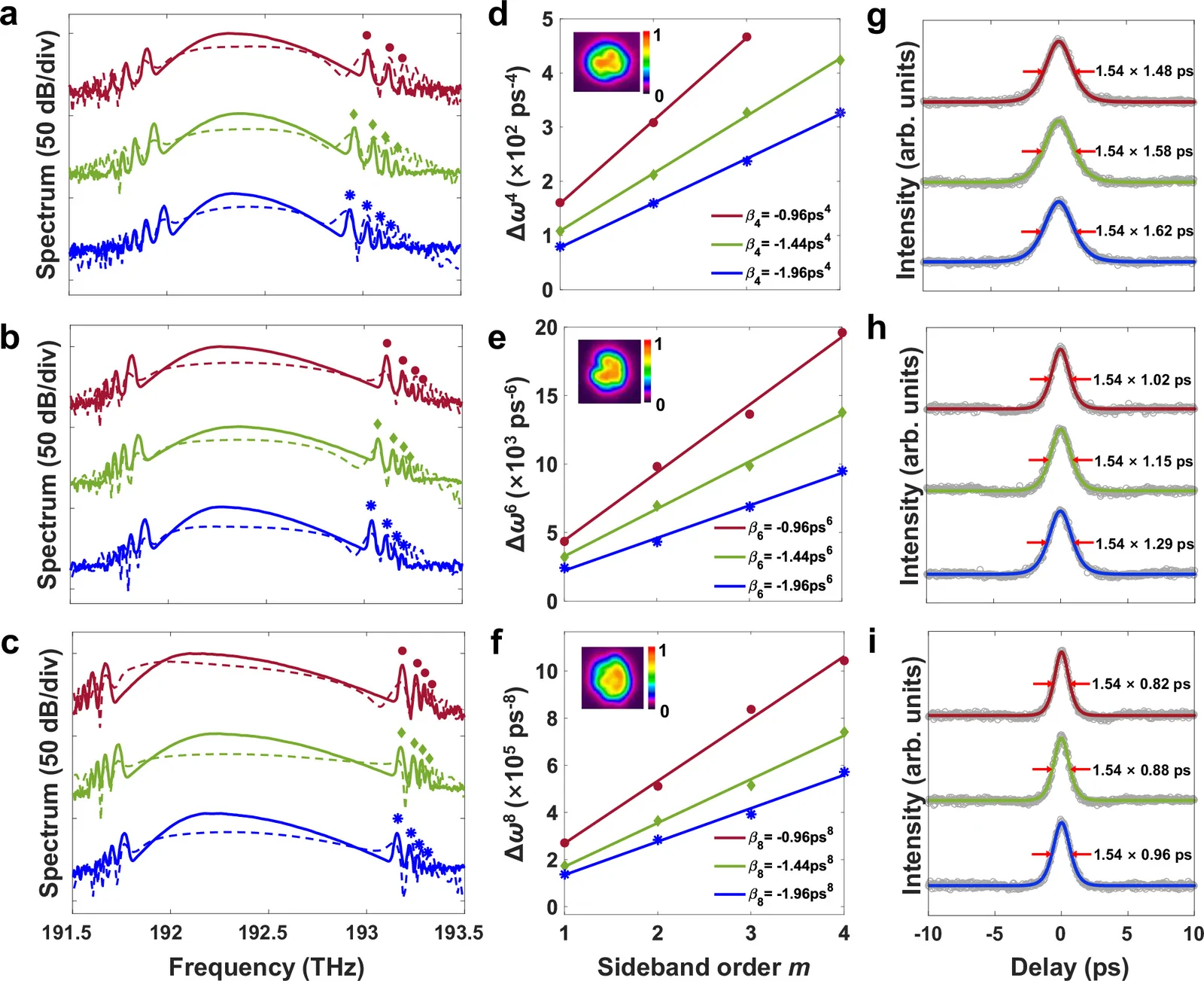
Characterization of spatiotemporally synchronized PHEOD solitons. **a–c** Typical optical spectra recorded under regimes dominated by pure *β*_4_ (**a**), *β*_6_ (**b**), and *β*_8_ (**c**). Solid and dashed curves show the experimental and simulated spectra, respectively. Solid symbols mark the sideband peak frequencies. **d–f** Analysis of sideband frequencies. Inset: beam profile. Distinct colors denote different intracavity dispersion values. **g–i** Measured ACs of the output pulses for the *β*_4_ (**g**), *β*_6_ (**h**), and *β*_8_ (**i**) regimes.

With the foundation of carefully characterized operation of a PHEOD soliton laser in each of the individual TMs established, we turn to experiments on MM operation. The laser is changed from the SM version to have all TMs connected in the TMDC, and the chromatic dispersion is set by the WaveShaper to be the same for each TM. The pump power is set to ~870 mW. With suitable adjustment of the PCs we observe an abrupt transition from fluctuating continuous-wave output to a train of ~1 ps pulses at the cavity repetition rate, which is accompanied by simultaneous changes in the output beam and the spectrum (see Supplementary Note 7). Apart from the output beam, which is clearly MM, the properties of the laser are qualitatively similar to those of SM mode-locking. However, there are important differences. Systematic experimental trends are summarized in Fig. 4. Based on the experimental observations and numerical simulations, we interpret the laser operation as the result of synchronization of the soliton pulse trains in the different TMs.

The spatially-integrated spectra (Fig. 4a–c) retain the central peak with clear sidebands, with two notable differences from the SM spectra: the sidebands are suppressed, and the spectra become asymmetric in the MM laser. The linear dependence of Δ*ω*^*k*^ on sideband index confirms that the MM fields are the result of the *k-*th order dispersion. However, in all higher-order dispersion regimes, the sidebands have lower intensities than their SM counterparts. This is clear from the comparison of Figs. 2, 4; the fraction of the energy in the first-order sidebands decreases by about a factor of 7 compared to the SM case (see Supplementary Note 6 for how the sideband energies are estimated). In addition, the central portion of the spectrum becomes asymmetric in the MM laser, with the peak of the spectrum shifting to lower frequency (Fig. 4a–c). This asymmetry increases with dispersion order.

Measurements of the pulse intensity profile exhibit clean single peaks within the ±10 ps observation window of the autocorrelator (Fig. 4g–i). The pulse duration exhibits systematic trends with dispersion. For a given dispersion order, the pulse duration increases gently with the magnitude of the cavity dispersion. On the other hand, the duration drops in steps, from ~1.5 ps to ~1.1 ps and ~0.9 ps, for *β*_4_, *β*_6_, and *β*_8_, respectively. The pulses are generally close to the transform limit, as determined by the zero-phase Fourier transform of the measured spectra: for *β*_4_ the MM pulses are about 50% beyond the transform limit, for *β*_6_ they are about 30% beyond the transform limit, and for *β*_8_ the duration is approximately equal to the transform limit.

**Fig. 5:**
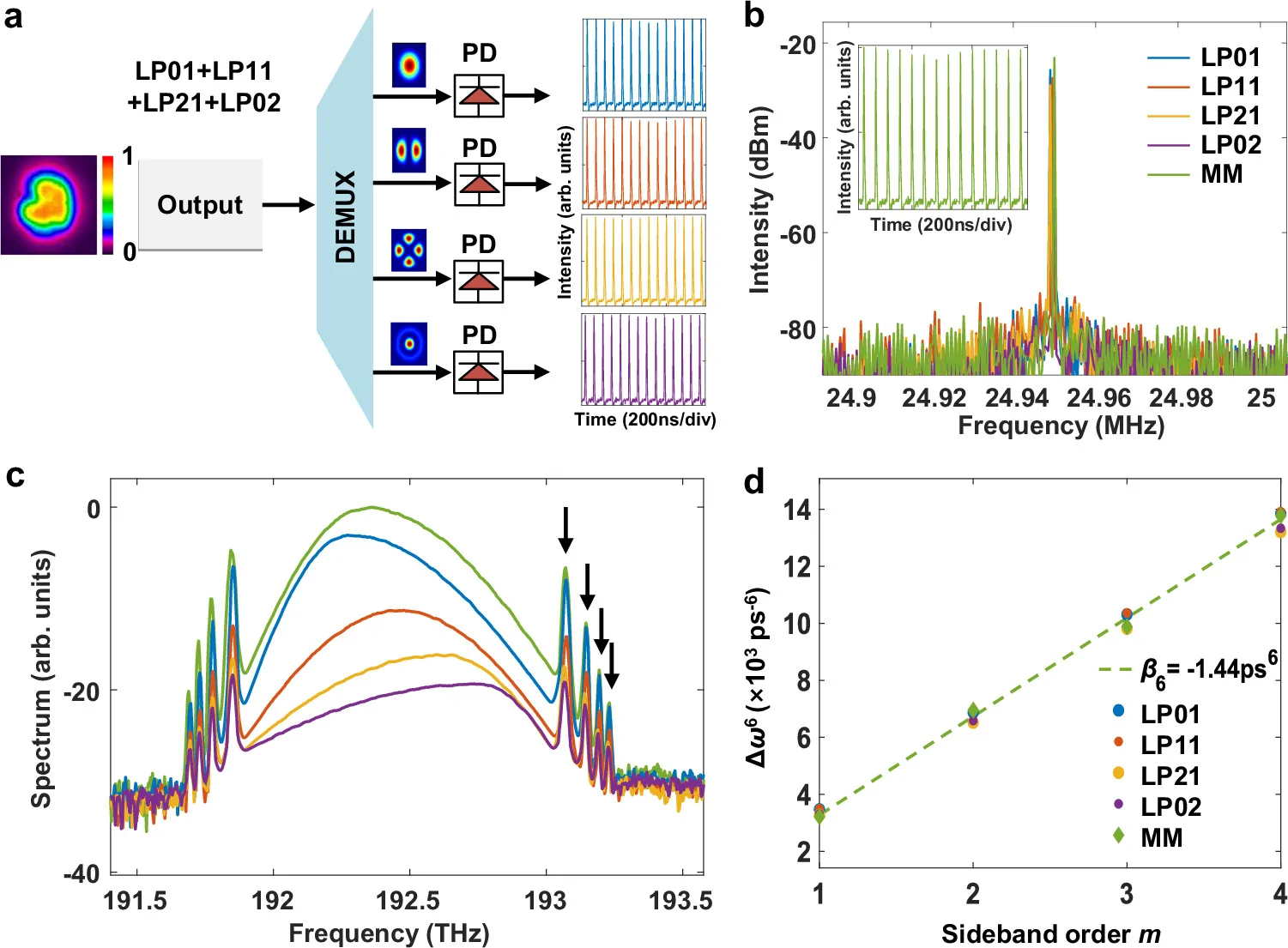
Mode-resolved characterization of spatiotemporally synchronized PHEOD solitons. All data were recorded at a net cavity dispersion of *β*_6_ = −1.44 ps^6^. **a** Schematic of the experimental setup for mode demultiplexing and verification of temporal synchronization. The output multimode (MM) beam (beam profile shown on the left) is demultiplexed into its transverse mode (TM) components (The mode profiles represent the corresponding intracavity modes, consistent with Fig. 1). Oscilloscope traces on the right display the synchronized pulse trains of the individual TM components. DEMUX, demultiplexer; PD, photodetector. **b** Radio-frequency (RF) spectra measured for each demultiplexed TM and the MM state. Inset: pulse train. **c** Comparison of the spectra of   demultiplexed TMs with the MM spectrum. **d** Quantitative analysis based on the sideband positions (indicated by black arrows in **c**).

The radio-frequency (RF) spectra provide some evidence of synchronization of the TMs. In SM operation, the repetition rates of the various TMs vary over about 10 kHz, directly corresponding to the measured group delays (see Supplementary Note 8). In MM operation, the repetition rates are pulled to within ~1 kHz of each other (an example is shown in Fig. 5b, which is discussed further below). That corresponds to a delay of ~ 1 ps, which is comparable to the pulse duration. The contrast against the background is about 55 dB, which is a bit below the 60 dB contrast of the SM mode-locked states.

Finally, the pulse energies are larger than the energies of SM solitons, but smaller than the sum of the energies in the individual modes (see Methods Tables 1, 2). Additional measurements under the same pump power are provided in Supplementary Note 11 which further characterize the pulse energies of the synchronized MM states.

Decomposition of the output pulses into constituent modes provides additional insight into the MM pulsed-lasing states. To accomplish this, we constructed a mode demultiplexing system (Fig. 5a) to separate the output beam into its four component TMs. Results obtained with pure sixth-order dispersion solitons (with net cavity dispersion of −1.44 ps^6^) are presented in Fig. 5, and similar data were recorded for the other dispersion orders. Temporal traces recorded with a fast detector and oscilloscope show that all of the modes display stable pulse trains. As mentioned above, the RF spectra of the individual modes are peaked within 1 kHz of each other. The asymmetry of the individual optical spectra varies systematically, with low-order modes shifted to lower frequencies and higher-order modes shifted to higher frequencies (Fig. 5c). Lastly, an analysis of the sideband positions further clarifies the relationship between the individual TMs and the synchronized MM state. As shown in Fig. 5d, the frequency shifts of the individual modes (scatter points) are distributed around the shifts of the MM state. Specifically, LP21 and LP02 modes fall slightly below the MM value, while the LP01 and LP11 modes show the opposite trend, which corresponds to the relative shifts of the sideband peaks observed in the mode-resolved spectra (Fig. 5c).

The output spectral and temporal profiles of the MM laser make clear that PHEOD soliton formation plays a major role in pulse shaping, but also indicate that strong nonlinear coupling among TMs occurs. In the absence of intermodal coupling, the individual TMs would essentially be separate laser cavities. In addition to their distinct repetition rates, their pulse trains would bear no temporal relationship with each other. The modal dispersion map provided by the TMDC could approximately equalize repetition rates, but the pulse trains in different TMs would be randomly delayed relative to each other. Nonlinear interactions are required to synchronize the fields in different TMs. The substantial observed differences between the SM and MM laser spectra (shown in Figs. 2, 4, 5c) can only be the result of intermodal nonlinear coupling processes.

The TMDC compensates for modal dispersion at the end of each round-trip of the cavity. The modal dispersion of the few-mode fibers separates pulses in different modes by 5–10 ps as they traverse the cavity, while even the large higher-order dispersion has only a weak effect on 1 ps pulses. Thus, in linear propagation, the pulses would separate by more than their duration, and nonlinear phase interactions occur over limited distances in the fiber. The modes are also coupled in the SA, where synchronized pulses lead to deeper saturation and less loss.

These assertions are supported by numerical simulations of linear pulse propagation in the cavity components as well as steady-state laser operation (see Supplementary Note 3). Good agreement between simulated and measured spectra (Fig. 4a–c) gives us confidence in drawing conclusions from the simulated pulse evolution (see Supplementary Note 2 for the simulation model). This confirms that the TMDC manages the modal dispersion and facilitates temporal overlap of pulses in the passive fiber between the output coupler and gain fiber, and in the SA. Even with the limited interaction length, cross-phase modulation pulls the modes together in time a bit, as does the interaction in the saturable absorber. Qualitatively, the shifts in frequency seen in Fig. 5c help to equalize the modal velocities. The suppression of the resonant sidebands that is observed in experiments with the MM laser is also seen in simulations, providing conceptual support for the possible mechanisms underlying the spectral changes, but is not as dramatic (see Supplementary Note 9).

To summarize the results of this section, we conclude that in MM operation, this laser generates spatially multimodal trains of synchronized PHEOD solitons. The synchronization is assisted by compensation of modal dispersion, which enhances nonlinear phase interactions between modes along with saturable absorption. We emphasize that the component solitons are not identical to solitons in individual TMs, owing to nonlinear coupling between the modes. Thus, the synchronized state is emergent; it develops through reorganization of the single-mode states. The abrupt transition in the spatial, temporal, and spectral properties upon entering the pulsed state is strong evidence for this. The change of beam profile when the output switches from continuous-wave to a highly periodic picosecond pulse train can only be explained by nonlinear interactions between TMs; i.e., spatiotemporal interactions.

## Discussion

We have demonstrated that PHEOD solitons in separate TMs of a fiber laser can be synchronized by controlling modal dispersion and nonlinear intermodal interactions. The TMDC technique facilitates this by spatially decoupling the TMs before applying phase shaping. Given that pulses in different modes do not completely overlap temporally, we would not describe the operation of the presented laser as STML, nor do we claim true phase-locking of the distinct TMs. Nevertheless, the results presented here represent a major step toward STML in PHEOD systems. Enhancing and controlling nonlinear interactions in future designs should position this platform for studies of STML in environments with higher-order dispersion. Spatiotemporal solitons in such environments may bear little resemblance to PHEOD solitons in SM waveguides.

Indeed, the laser operation reported above shares some characteristic features of STML. Single spatiotemporal pulses emerge spontaneously from spatially- and temporally-fluctuating fields under suitable conditions, and the onset of the synchronized state is associated with a reorganization of the excitations of the MM field. TMs with repetition rates of 25 MHz are synchronized to the 1-kHz level, which is comparable to results in STML^18^. The generated spatiotemporal wave packets have nearly transform-limited temporal profiles, which typically requires phase-locking of modes.

STML refers to the phase-locking of transverse and longitudinal modes of a laser to create a wave packet that traverses the cavity as a coherent object. In the laser described here, on each round trip, wave packets ~1 ps in duration separate by 5 to 10 ps owing to the local modal dispersion before being recombined by the modal dispersion map. The limited temporal overlap hinders strong phase-sensitive interactions, although there is clear evidence of some nonlinear interactions. With larger chromatic dispersion or better control of modal dispersion, it will be interesting to explore STML with higher-order dispersion in the future. Normal dispersion may offer advantages in this regard, because it supports longer, chirped pulses.

While we believe that the architecture described here will enable investigation of a variety of new nonlinear wave phenomena, some technical limitations remain. First, due to current pulse shaper hardware constraints, we apply a globally uniform dispersion phase profile to all TMs. However, the physical spatial separation of TMs provided by our architecture is essential for this operation. Although this simplified ‘unified mask’ approach is enough to generate PHEOD solitons, the current hardware limitations prevent precise compensation for the minor residual dispersion differences between TMs. Future implementations might allow operation at much smaller values of the PHEOD, which would allow exploration of distinct propagation regimes. Second, the durations of PHEOD solitons currently remain relatively long. It would be very interesting to push into the 100 fs regime to explore the increase of pulse energy predicted by the steep energy-width scaling. This will require more precise management of modal dispersion in the cavity. However, these limitations are mainly associated with the present proof-of-concept implementation. Future improvements could include high-resolution, multi-channel shaping schemes for accurate independent phase control, as well as high-damage-threshold static components and high-precision delay compensation devices to overcome the energy and walk-off bottlenecks.

Finally, the laser cavity design introduced here is versatile and can be applied to other MM platforms, such as MM solid-state lasers or integrated photonic circuits. The capability to independently manipulate temporal phase and spatial distribution enables new possibilities for generating light with customized spatiotemporal structures. Future research might explore the generation of custom spatiotemporal wave packets or synthesizing vortex beams with specific spatiotemporal orbital angular momentum^35^.

## Methods

### Laser cavity

The experimental setup for generating spatiotemporally synchronized PHEOD solitons is shown in Fig. 1a. The gain fiber used in the laser is an FM-EDF with a length of 1.5 m. The laser was continuously pumped and operated at room temperature. All other cavity components are made with four-mode FMF, which supports the LP01, LP11, LP21, and LP02 modes at 1550 nm. The mode MUX/DEMUX adopts a mode-selective coupler cascade structure (insertion loss is less than 0.4 dB and intermodal crosstalk isolation is > 19 dB, see Supplementary Note 10 for detailed characterization). The differential group delays between the fundamental mode (LP01) and the higher-order modes (LP11, LP21, and LP02) are measured by time-of-flight (see Supplementary Note 8). According to the fiber parameters, each meter of SMF can compensate for a delay of 4.829 ns. Therefore, at the demultiplexing end of the mode DEMUX, the relative SMF lengths of the LP01, LP11, and LP21 channels are adjusted by 2.7, 2.1, and 1.1 mm, respectively, to achieve the balance of intermode dispersion. 20% of the intracavity energy is extracted via an OC for extracavity characterization. In the system, all devices are fusion-spliced with no core offset, avoiding unintended intermode coupling.

For the output pulses, the optical spectrum is measured using an optical spectrum analyzer (OSA, Anritsu MS9710B) with a resolution of 0.2 nm. The optical pulses are converted to electrical signals via an MM photodetector (PD, Conquer KG-PT-20G-A-MM-FC) and then recorded in the time domain using an oscilloscope (OSC, Tektronix DSA72004C). The RF spectrum is characterized by a signal analyzer (Keysight N9020A MXA). The AC trace is obtained using an autocorrelator (Avesta AA-20DD), while the output beam profile is captured by an infrared camera (Ophir SP90606).

### Laser operation and pulse energy characterization

The starting mechanism for pulse generation relies on nonlinear polarization  rotation. To establish a stable PHEOD state, the target high-order dispersion mask is first applied through the WaveShaper. Subsequently, after raising the pump power above the threshold, the PCs are adjusted until a stable pulse train is observed.

For the mode-selective generation of PHEOD solitons, the operating pump power is uniformly maintained at 1.8 W. The measured pulse energies under a fixed dispersion magnitude of −1.44 ps^*k*^ are detailed in Table 1. For the generation of four-mode PHEOD solitons, the mode-locking is initiated at a lower pump threshold of 870 mW, and the operating pump power is fixed at 1.6 W during characterization. The corresponding pulse energies across various dispersion orders and magnitudes are summarized in Table 2. The pulse energies are higher than the SM counterparts, despite operating at a lower pump level. As shown in both tables, the pulse energy decreases at higher dispersion orders and larger dispersion magnitudes under a fixed pump power.

**Table 1:** Pulse energy (in pJ) of PHEOD soliton in individual TMs

	LP01	LP11	LP02	LP21
k = 4	101.0	87.0	77.8	72.6
k = 6	95.4	77.0	75.8	70.1
k = 8	88.6	73.7	69.3	66.5

**Table 2:** Pulse energy (in pJ) of four-mode PHEOD solitons

	−0.96ps^*k*^	−1.44 ps^*k*^	−1.96 ps^*k*^
k = 4	206.8	185.2	159.5
k = 6	195.6	159.3	115.4
k = 8	180.8	125.1	66.1

### Sideband analysis

To confirm that the output pulses are indeed governed by specific high-order dispersion mechanisms, and to verify the net intracavity dispersion, we compare the measured and predicted sideband frequencies.

In our experiments, the intracavity *β*_2_ values are adjusted to small positive values, while *β*_3_ is suppressed to near-zero values, ensuring that the dispersion is dominated by the artificially introduced *k*-th order dispersion terms (*k* = 4, 6, 8). Under these conditions, the phase evolution of the linear dispersive waves is dictated primarily by the *k*-th order term, whereas the PHEOD soliton experiences a uniform nonlinear phase shift across its entire bandwidth. Consequently, the frequency shift Δ*ω*_*m*_ of the *m*-th spectral sideband relative to the central frequency obeys the following analytical relationship:

1 $$\Delta {\omega }_{m}=\pm \frac{1}{\tau }{\left[k!\left(\frac{2\pi m{\tau }^{k}}{|{\beta }_{k}|L}-{C}_{k}\right)\right]}^{1/k}$$

where *τ* denotes the pulse duration, *L* represents the laser cavity length, *β*_*k*_ is the dominant *k*-th order dispersion coefficient, and *C*_*k*_ is a constant associated with the soliton waveform. In analyzing the experimental data, we measure the central positions of the spectral sidebands across different modes. We then plot the functional relationship (specifically, the frequency shift raised to the power of *k*) against the sideband index *m*, followed by a linear fit. A high degree of linearity in the data points serves as evidence that the soliton formation is indeed governed by pure *k*-th order dispersion.

## Supplementary information

Supplementary Information

Transparent Peer Review file

## Source data

Source Data

## Data Availability

The data generated in this study are provided in the Source Data file.

## Code availability

The code used in this manuscript is available from the corresponding author upon request.
