# Supplementary material for: Spatiotemporally synchronized pure high-even-order dispersion solitons in multimode fiber lasers
Source: Nat Commun. 2026 Oct 3;17:11307. doi: 10.1038/s41467-026-78194-5 (PMC13724690; doi:10.1038/s41467-026-78194-5)
Supplement: Supplementary file 1 [file 41467_2026_78194_MOESM1_ESM.pdf]

Supplementary Information for:

Spatiotemporally synchronized pure high-even-order dispersion solitons in multimode fiber lasers

Author Information

Wenqi Ma[1], Xiuquan Li[1], Canyi Deng[1], Caishan Pang[1], Rui Nie[1], Frank W. Wise[3] and Guijun Hu[1,2]*

[1]College of Communication Engineering, Jilin University, Changchun 130012, China

[2]Jilin Provincial Key Laboratory of Specialty Optical Fiber Fabrication and Application, Changchun 130012, China

[3]School of Applied and Engineering Physics, Cornell University, Ithaca, New York 14850, USA

Email: hugj@jlu.edu.cn

This file includes:

Supplementary Notes 1 to 11

Supplementary Figures 1 to 14

Supplementary Table 1 to 2

Supplementary References

Supplementary Note 1: Dispersion compensation strategy

Due to pulse-shaper constraints, we adopted a uniform dispersion profile across all TM channels. To implement the maximum-dispersion-based compensation strategy, we select the TM exhibiting the largest absolute value of intrinsic intracavity β_2 as the reference benchmark. By loading a specific phase mask onto the SLM, we compensate the net cavity β_2 of this benchmark mode to a near-zero positive value. Given that the magnitude of the intrinsic anomalous dispersion for all other modes is lower, this inherently guarantees that the net β_2 for all oscillating modes remains strictly within the normal dispersion regime. The applied phase distribution can be expressed as:

$$\Phi(\omega) = L\left(\sum_{n=2}^{3}\frac{\beta_n\left(\omega-\omega_0\right)^n}{n!}+\frac{\beta_k\left(\omega-\omega_0\right)^k}{k!}\right) \tag{1}$$

where β_n represents the n-th order dispersion coefficient (for n=2, 3).

In this study, for the SMF-28e, the chromatic dispersion parameter is 17 ps (nm•km)$^{-1}$ (uncertainty: 0.02%) and β_3 = 0.12 ps^3/km (uncertainty: 0.4%)[1]. For the FMF (YOFC, FM2011-B), the parameters are based on the specifications provided by the manufacturer: the chromatic dispersion parameters for the LP_{01}, LP_{11}, LP_{21}, and LP_{02} modes are 21.9, 22.5, 22.0, and 21.8 ps (nm•km)$^{-1}$, respectively (uncertainty < 0.08%), with corresponding β_3 values of 0.1501, 0.1504, 0.1481, and 0.1554 ps^3/km, respectively (uncertainty < 3%). To compensate for the intracavity dispersion, the β_2 and β_3 values applied via the SLM are set to 0.1826 ps^2 and -0.0012 ps^3, respectively. The resulting net residual dispersion for each TM, alongside their intrinsic cavity dispersion and the targeted high-order dispersion values, are summarized in Supplementary Table 1. The second term on the right-hand side of Supplementary Equation (1) represents the dominant negative high-even-order dispersion introduced to govern the pulse dynamics. This introduced dispersion term plays a decisive role because the intrinsic high-order dispersion of fibers is negligibly small (e.g., intrinsic β_4 is ~10^{-3} ps^4/km[1], and β_6, β_8 are even smaller).

Supplementary Table 1 | Summary of intrinsic, residual, and targeted dispersion values for each TM

TM	Intrinsic cavity β_2 (ps^2)	Net residual β_2 (ps^2)	Intrinsic cavity β_3 (ps^3)	Net residual β_3 (ps^3)	Targeted β_k (ps^k)
LP01	-0.1773	+0.0053	+0.00118	-0.00002	-0.96/ -1.44/ -1.96
LP11	-0.1816	+0.0010	+0.00120	0	
LP21	-0.1778	+0.0048	+0.00117	-0.00003	
LP02	-0.1761	+0.0065	+0.00113	-0.00007	

Supplementary Note 2: Numerical observation of the spatiotemporally synchronized PHEOD soliton generation

To investigate the formation mechanism of PHEOD solitons in MM fiber lasers, we developed a numerical simulation model based on the generalized multimode nonlinear Schrödinger equation (GMMNLSE). This model comprehensively describes the complex dynamics of pulse evolution within MM fibers, incorporating dispersion, Kerr nonlinearity, gain saturation, and loss. The governing equation is expressed as[2]:

$$\begin{aligned}\partial_z A_p(z,t) &= i\left(\beta_0^{(p)} - \beta_0^{(0)}\right)A_p - \left(\beta_1^{(p)} - \beta_1^{(0)}\right)\frac{\partial A_p}{\partial t} + i\sum_{n\geq 2}\frac{\beta_n^{(p)}}{n!}\left(i\frac{\partial}{\partial t}\right)^n A_p \\ &+ i\frac{n_2\omega_0}{c}\sum_{l,m,n} S_{plmn}^K A_l A_m A_n^* + \mathcal{F}^{-1}\left\{\frac{g_0 f(\omega)}{2\left(1+\sum_{n=1}^{N}\int |A_n|^2 dt / E_{sat}\right)} A_p(\omega,t)\right\}\end{aligned} \quad (2)$$

where $A_p(z,t)$ represents the mode field envelope of mode *p*, and $\beta_n^{(p)}$ denotes the *n*-th order dispersion of mode *p*. n_2 is the nonlinear refractive index of the fiber medium, ω_0 is the central angular frequency, and c is the speed of light. S_{plmn}^K is the coupling coefficient derived from the Kerr nonlinear overlap integrals between modes, N is the total number of modes, $f(\omega)$ represents the Gaussian-distributed gain spectrum, E_{sat} denotes the saturation energy of the Erbium-doped fiber, and g_0 is the small-signal gain (set to 0 for passive fibers). To simplify the model, a simplified Gaussian gain profile was utilized, and stimulated Raman scattering and self-steepening effects were neglected. Based on experimental observations, the four dominant linearly polarized modes—LP01, LP11, LP21, and LP02—were selected for the simulation, and all fiber parameters employed were consistent with the experimental setup.

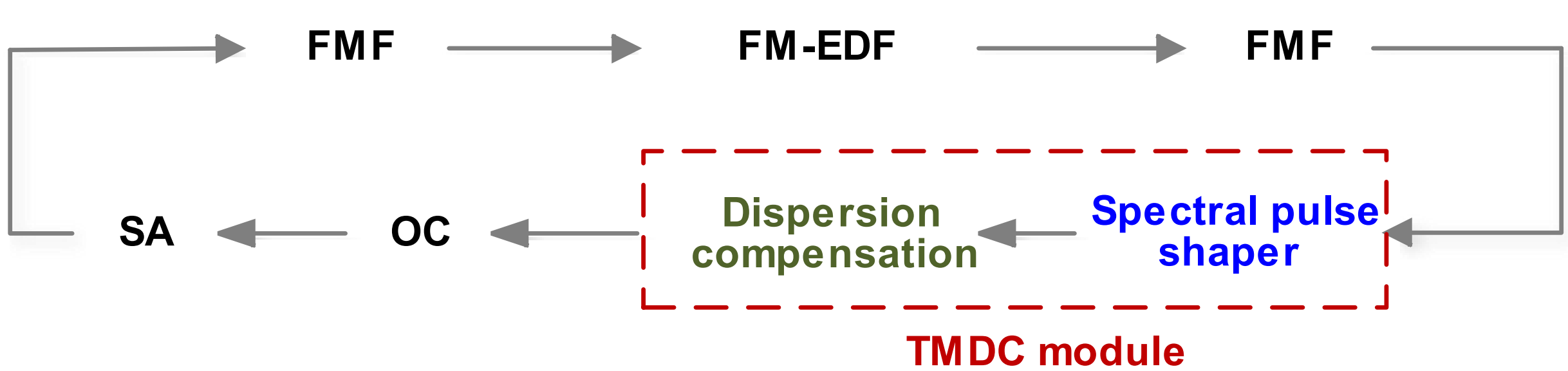

Supplementary Fig. 1 | Schematic diagram of the numerical simulation for generating spatiotemporally synchronized PHEOD solitons. FMF, few-mode fiber; FM-EDF, few-mode erbium-doped fiber; TMDC, transverse mode division control; OC, output coupler; SA, saturable absorber.

Based on the aforementioned theoretical framework, the simulated laser cavity model primarily comprises a few-mode erbium-doped fiber (FM-EDF), a few-mode fiber (FMF), a TMDC module, an output coupler (OC), and a saturable absorber (SA), with the schematic structure depicted in Supplementary Fig. 1. Crucially, the TMDC module serves as the core component for realizing PHEOD soliton generation. In the simulation, the model first spatially decomposes the MM optical field into independent TM components. Then, group velocity mismatch (GVM) compensation is introduced for each mode to ensure the synchronization of the distinct TMs. Subsequently, a spectral pulse shaper is employed to apply phase modulation to each synchronized mode component. By loading specific phase masks in the frequency domain, we introduce the target high-order dispersion (β_k, $k \geq 4$) while compensating the accumulated β_2 and β_3 of the fiber. The phase function is defined as follows:

$$\Phi_p(\omega) = L_1 \sum_{n\geq 2} \frac{\beta_{p,n}\left(\omega-\omega_0\right)^n}{n!} + L_2 \sum_{n\geq 2} \frac{\beta'_{p,n}\left(\omega-\omega_0\right)^n}{n!} + L_{3,p} \sum_{n\geq 2} \frac{\beta''_{p,n}\left(\omega-\omega_0\right)^n}{n!} \tag{3}$$

where L_1 and L_2 denote the lengths of the gain fiber and the passive fiber, respectively, while L_3 represents the length of the compensating fiber for the p-th mode. This configuration ensures that, for each intracavity round trip, the low-order dispersion of all modes is compensated, leaving the pulse propagation dominated by high-even-order dispersion. Subsequently, the dispersion-managed mode components are recoupled into an FMF, where 20% of the energy is extracted via an output coupler. The remaining energy propagates into the SA. To characterize the nonlinear loss dynamics of the MM optical field within the SA, we employed a mode-multiplexed three-dimensional SA model that accounts for the discrepancies in effective mode area:

$$T_{p,SA} = \sqrt{1 - q \cdot \exp\left(-\frac{\left|E_p\right|^2}{P_{sat} / A_{p,eff}}\right)} \tag{4}$$

where $T_{p,SA}$ represents the transmittance of the p-th mode, q denotes the modulation depth. P_{sat} is the saturation power, and $A_{p,eff}$ corresponds to the effective mode area of mode p. The simulation was initiated using a Gaussian pulse with a total energy of 3 nJ, uniformly distributed across the four simulated modes. By iteratively solving the equations and dynamically adjusting g_0 to emulate the pumping level, the laser field was allowed to evolve into a steady-state mode-locked operation.

To validate the experimental observations, we simulated the generation of high-order solitons under various dispersion orders (k = 4, 6, 8) and different dispersion magnitudes that match the experimental conditions. To facilitate comparison between simulation and experiment, the simulated spectra are presented alongside the experimental data in Fig. 4. For a mode-resolved analysis, we extracted the simulated fields specifically under the pure sixth-order dispersion regime (β_6 = -1.44 ps^6).

Supplementary Fig. 2a shows the simulated spectra of the resolved LP01, LP11, LP21, and LP02 components, together with the total MM output. The individual TM spectra are not identical, but their sideband positions remain closely related to those of the synchronized MM state. The central spectral envelopes show different amplitudes and frequency shifts, indicating that the component modes are reorganized during synchronized MM operation rather than behaving as independent SM solitons. The sideband-position analysis in Supplementary Fig. 2b further clarifies the relationship between the individual TMs and the synchronized MM output. The frequency shifts of the resolved TM sidebands are distributed around those of the MM state. For all resolved TMs and for the MM output, $\Delta\omega^6$ remains approximately linear with the sideband order m.

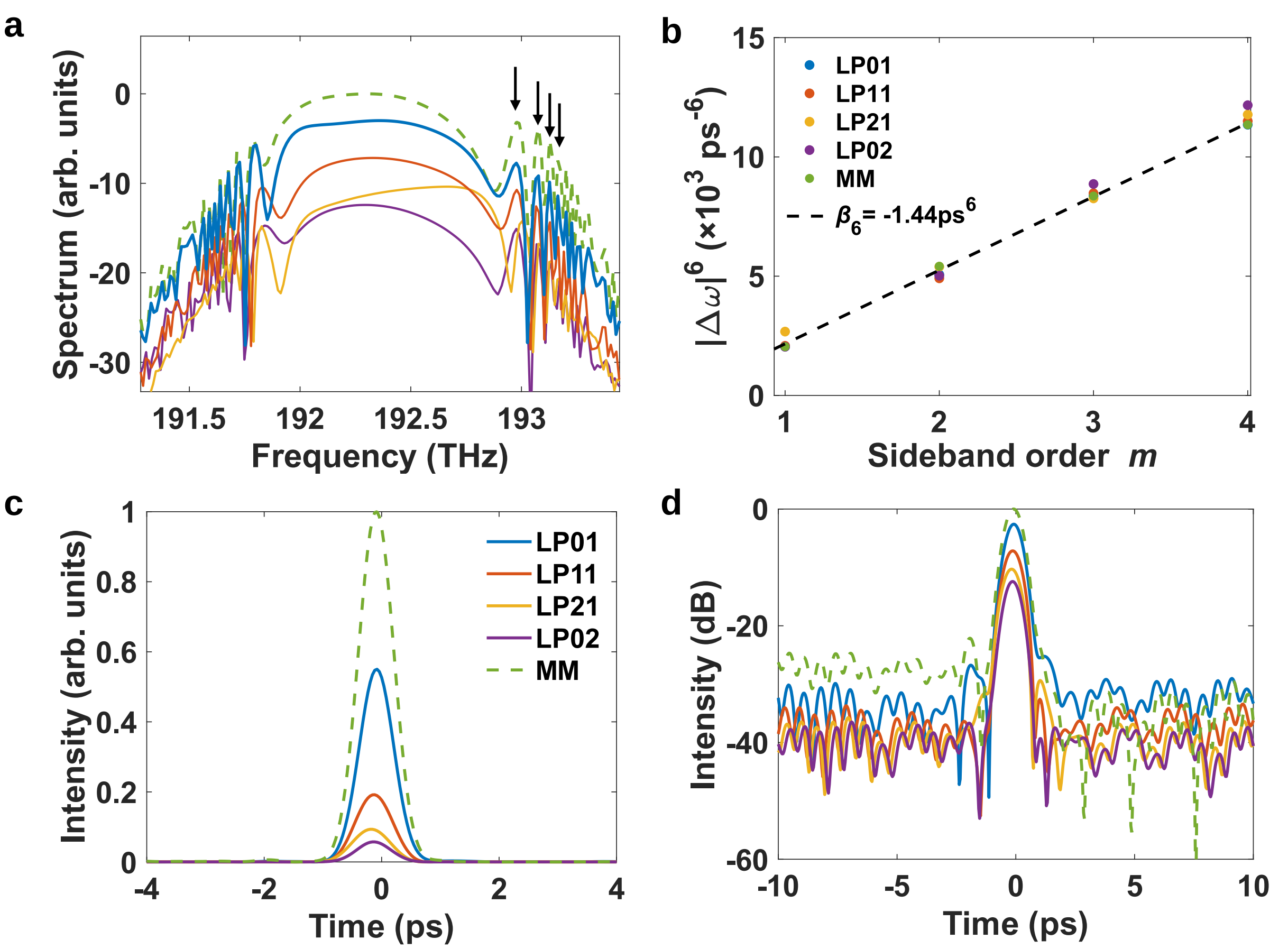

Supplementary Fig. 2 | Simulated mode-resolved characterization of four-mode PHEOD solitons. a, Simulated optical spectra of the resolved TMs (LP01, LP11, LP21, LP02) and the MM output at a net intracavity dispersion of β_6 = -1.44 ps^6. **b,** Quantitative analysis of the Kelly sideband positions for each constituent mode and the MM output. Dashed black lines indicate theoretical predictions. **c, d,** Simulated temporal intensity profiles of the demultiplexed pulses in linear (c) and logarithmic (d) scales.

The simulated temporal intensity profiles are shown in Supplementary Fig. 2c,d. In the linear-scale traces (Supplementary Fig. 2c), the pulses in different TMs overlap within the main pulse window, indicating the formation of a synchronized pulsed state. The different peak amplitudes reflect the unequal modal energy distribution in the simulated synchronized MM state. In the logarithmic-scale traces (Supplementary Fig. 2d), weak oscillatory temporal tails can be observed around the main pulse. These oscillatory tails are a characteristic feature of PHEOD soliton solutions and are more visible in the simulations than in the experiment because they are not obscured by measurement noise or limited dynamic range[3,4].

Supplementary Note 3: Numerical simulation of intracavity modal walk-off and the synchronization mechanism

To clarify the intracavity MM dynamics, we simulated pulse propagation over multiple cavity round trips (RTs) using the GMMNLSE. The modal walk-off was quantified by tracking the temporal center of gravity of each TM relative to that of LP01.

Supplementary Fig. 3a shows the steady-state walk-off evolution over the last four RTs. During propagation through the active and passive FMFs, the higher-order modes accumulate temporal delays relative to LP01 owing to the modal GVM, with LP02 exhibiting the largest walk-off. At the TMDC module, the accumulated modal walk-off is compensated at the end of each RT, so that the pulses are brought back close to temporal overlap before entering the following cavity section. After the TMDC module, the pulses propagate through a short pigtail fiber before reaching the output coupler (OC), where only a small additional walk-off of approximately 0.12–0.39 ps is accumulated.

To distinguish the role of modal compensation from that of nonlinear interactions, we compared the early-stage evolution under two different pulse-energy conditions. In the normal operating regime, as shown in Supplementary Fig. 3b, small initial temporal offsets were deliberately introduced, while the TMDC compensation was kept fixed. Although a slight residual mismatch remains after each RT, the relative delays do not grow without bound. Instead, the pulse trains evolve toward a repeatable synchronized state with small residual offsets. This behavior indicates that the linear modal-dispersion map provided by the TMDC module is the primary mechanism that prevents large walk-off accumulation, while nonlinear interactions, including cross-phase modulation and the intensity-dependent loss of the SA, further assist the temporal pulling and stabilization of the modes.

For comparison, Supplementary Fig. 3c shows the evolution when the initial pulse energy is reduced to one-tenth of the normal value. In this low-energy case, the nonlinear effects and SA gating are much weaker. The residual walk-off then accumulates over successive RTs instead of being pulled into a stable periodic pattern. This comparison shows that TMDC compensation alone can largely reduce the modal delay mismatch, but the stable synchronized pulsed state observed in the full laser requires the combined action of the modal-dispersion map, nonlinear intermodal interactions, and saturable absorption.

These simulations help explain the synchronized MM operation observed in the experiment. The TMDC module compensates the accumulated modal dispersion once per RT and enhances temporal overlap in selected cavity sections, especially near the SA. Between compensation points, however, the pulses still separate by several picoseconds in the FMFs, so the nonlinear interaction length is limited. Therefore, the simulated state should be understood as a train of spatiotemporally synchronized PHEOD solitons, rather than as a fully developed conventional spatiotemporal soliton. The component solitons are reorganized by

intermodal coupling and saturable absorption, but they are not simply identical copies of the independently generated single-mode PHEOD solitons.

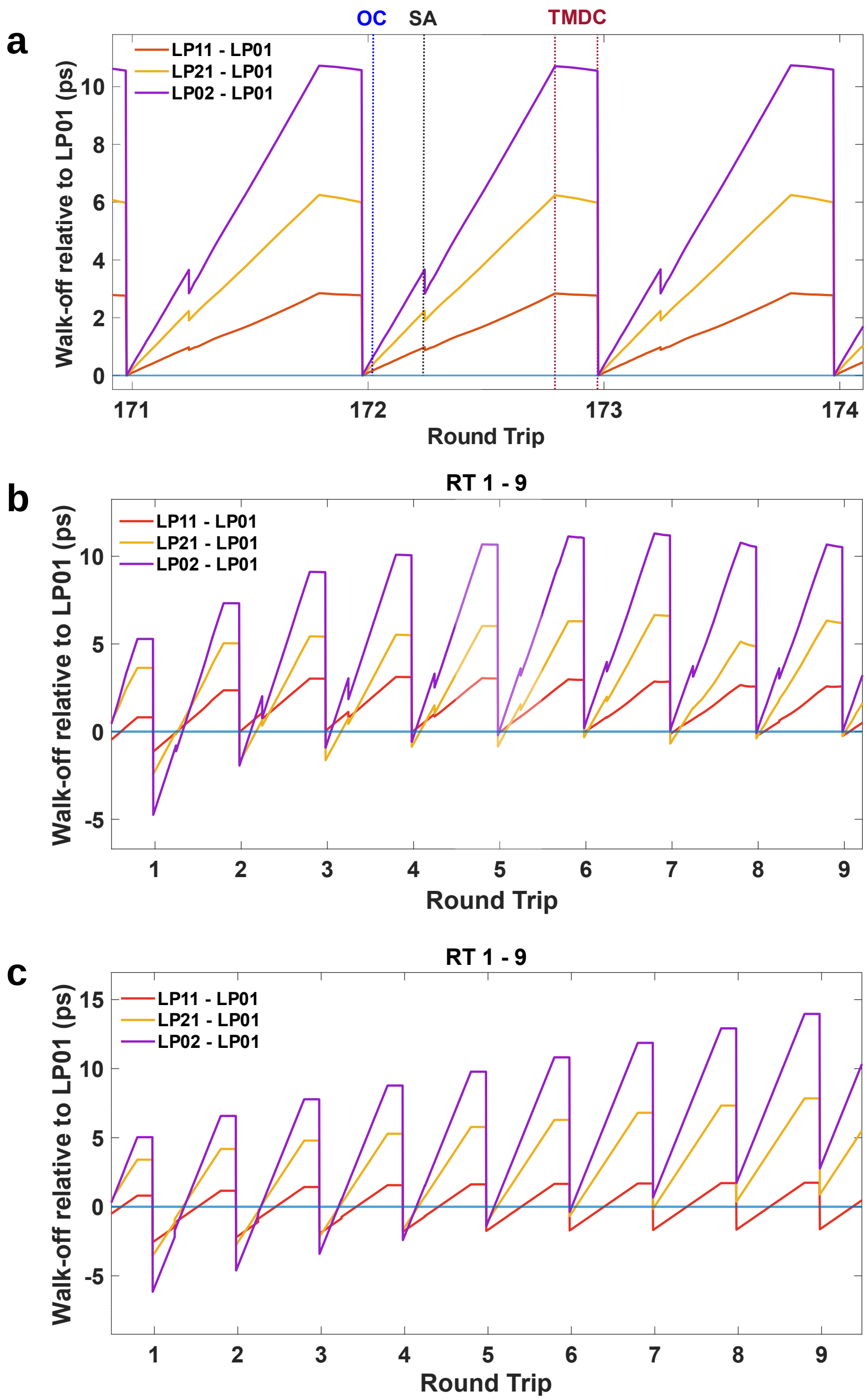

Supplementary Fig. 3 | Numerical simulations of intracavity modal walk-off. a, Steady-state walk-off evolution over the last 4 round trips (RTs). **b,** Walk-off evolution over the first 9 RTs in the nonlinear regime, showing self-correction towards stability. **c,** Walk-off evolution over the first 9 RTs in the low-energy regime, showing continuous accumulation. OC, output coupler; SA, saturable absorber; TMDC, transverse mode division control.

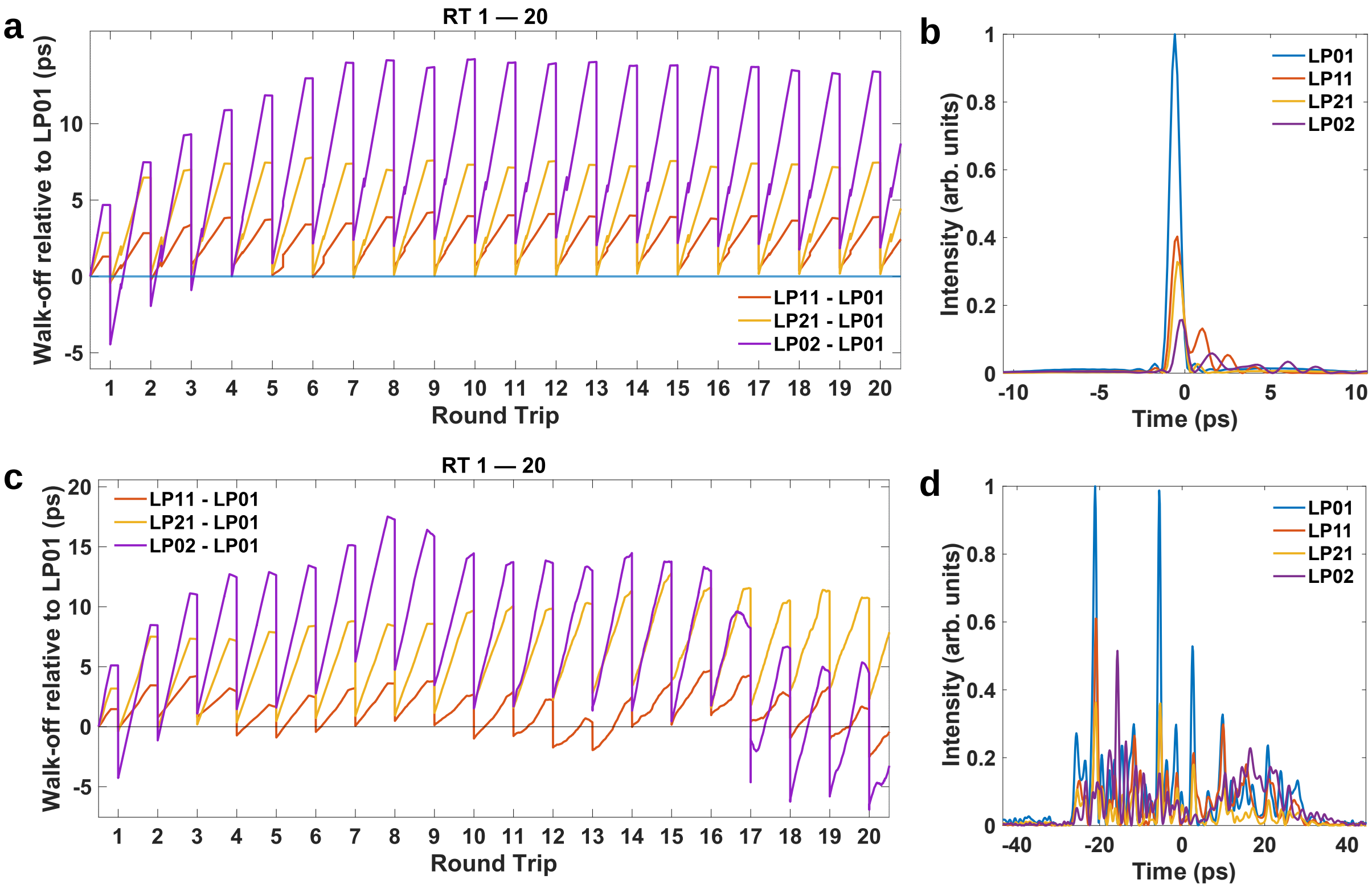

Supplementary Fig. 4 | Controlled simulations with selected nonlinear and SA effects removed. a, b, Intracavity relative modal walk-off over 20 round trips and the corresponding temporal profiles when the intermodal nonlinear terms (XPM and FWM) are removed while SPM is retained. **c, d,** Corresponding intracavity relative modal walk-off and temporal profiles when the SA effect is removed. XPM, cross-phase modulation; FWM, four-wave mixing; SPM, self-phase modulation; SA, saturable absorber.

To further examine the roles of the Kerr nonlinearity and the SA, we performed two additional controlled simulations. First, we removed the intermodal nonlinear terms (XPM and FWM) and the corresponding results are shown in Supplementary Fig. 4a, b. Although the TMDC still periodically resets the accumulated modal walk-off, a substantial relative delay remains between LP02 and LP01, and the temporal profiles develop pronounced satellite and multi-peak structures. As a result, the system does not recover the stable synchronized state obtained in the full model (Supplementary Fig. 2c). We then removed the SA effect, and the results are shown in Supplementary Fig. 4c,d. In this case, the relative delays among the modes become markedly more irregular during the round-trip evolution, and the temporal profiles undergo severe broadening and fragmentation, so that no stable localized pulsed state is obtained. These results show that periodic linear compensation alone is insufficient; both intermodal Kerr nonlinearity and the SA contribute to the formation and stabilization of the synchronized state.

Supplementary Note 4: Quantitative comparison of dispersion contributions and sensitivity analysis of PHEOD solitons

To evaluate whether the applied high-even-order dispersion dominates over the residual low-order dispersion within the spectral range relevant to soliton formation, we calculated the spectral phase contributions from both the residual low-order terms (k = 2, 3) and the applied PHEOD terms (k = 4, 6, 8). The absolute phase contribution of the k-th order dispersion is defined as:

$$|\Phi_k(\Omega)| = \frac{1}{k!}|\beta_k|\Omega^k \tag{5}$$

where β_k is the k-th order dispersion coefficient, and Ω is the angular frequency offset from the central frequency. Using the largest residual low-order dispersion values measured in the experiment (β_2= 0.0065 ps^2 and β_3 = -0.00007 ps^3), we plotted the phase magnitudes on a logarithmic scale as a function of Ω. As shown in Supplementary Fig. 5, within the sideband region, the applied high-even-order contributions exceed the residual low-order contributions and increase much more rapidly with frequency offset, reaching values of 10^2–10^3 rad at larger offsets. This large separation indicates that the spectral phase accumulated in the sideband region is dominated by the applied high-even-order dispersion, with much smaller contributions from the residual β_2 and β_3.

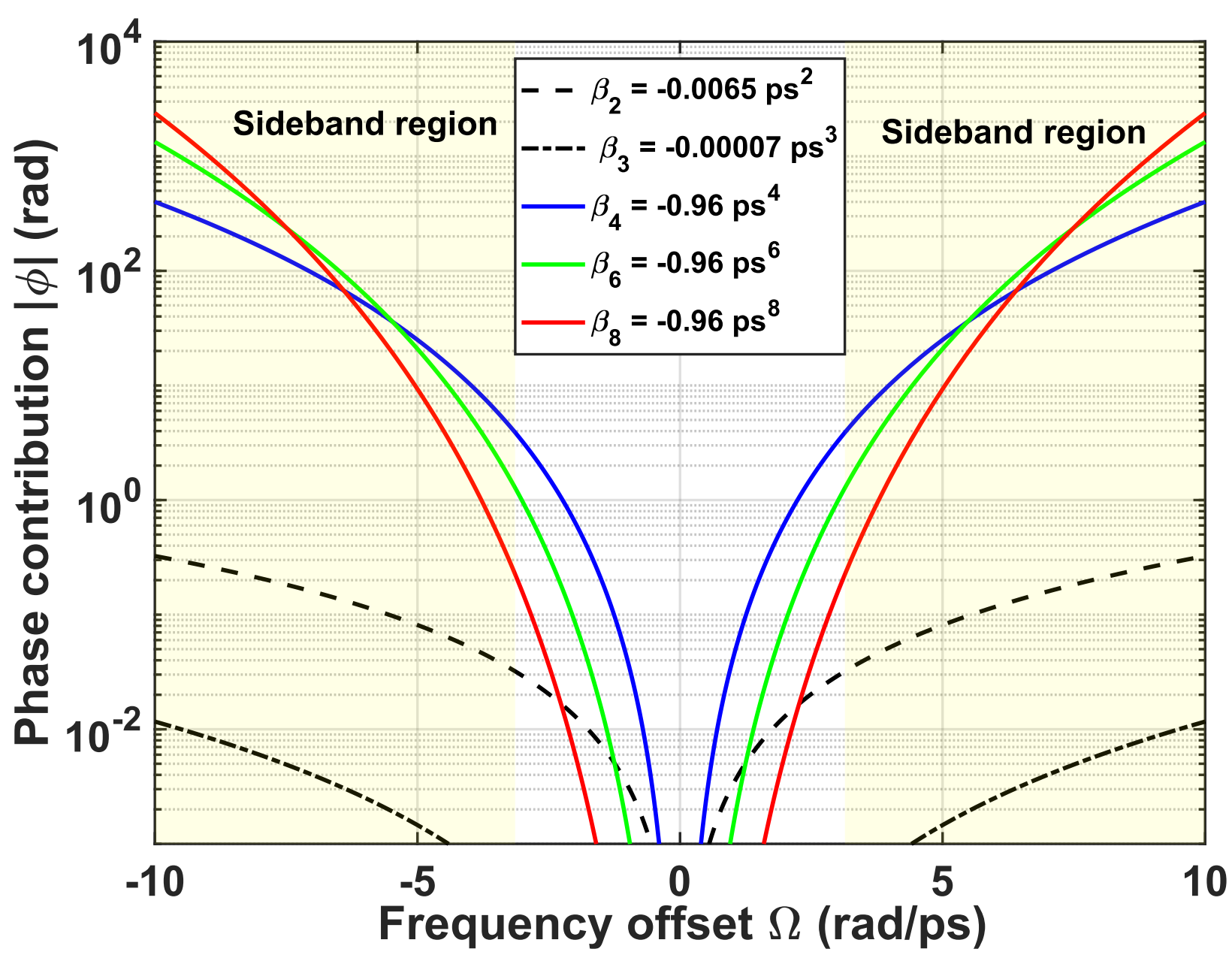

Supplementary Fig. 5 | Quantitative comparison of spectral phase contributions from different dispersion orders. The dashed and dash-dotted black lines represent the phase shifts induced by the worst-case residual low-order dispersion (β_2 = 0.0065 ps^2 and β_3 = -0.00007 ps^3), respectively. The solid lines

represent the applied PHEOD terms (β_4, β_6, β_8 = -0.96 ps^k). The shaded yellow areas highlight the critical sideband generation regions (|Ω| > 3.14 rad/ps).

We further evaluated the group delay introduced by each dispersion order. The group delay relative to the central frequency is obtained by differentiating the spectral phase:

$$\Delta\tau(\Omega) = \frac{d\Phi}{d\Omega} = \sum_{k\geq 2} \frac{\beta_k}{(k-1)!}\Omega^{k-1}. \quad (6)$$

Therefore, the contribution from an individual k-th-order dispersion term is:

$$\Delta\tau_k(\Delta f) = \frac{\beta_k}{(k-1)!}\left(2\pi\Delta f\right)^{k-1}, \quad (7)$$

where $\Omega = 2\pi\Delta f$ and $\Delta f = f - f_0$. Using this expression, we plotted the group delay contributions over the experimentally measured spectral ranges for the β_4, β_6, and β_8 regimes, as shown in Supplementary Fig. 6. The largest residual low-order dispersion values measured among the participating TMs, β_2 = 0.0065 ps^2 and β_3 = -0.00007 ps^3, are compared with the group delay contributions of the applied β_4, β_6, and β_8, for which β_k = -1.96 ps^k (k = 4,6, and 8). The corresponding experimental spectra are independently normalized and overlaid to indicate the actual frequency ranges occupied by the central spectra, spectral wings, and resolved resonant sidebands.

As shown in Supplementary Fig. 6, the applied high-even-order group delay contributions increase much more rapidly with frequency offset than the residual low-order contributions. For the β_4, β_6, and β_8, the intended high-even-order terms dominate in the experimentally observed spectral-wing and resolved resonant-sideband regions, where the characteristic PHEOD spectral features occur. In the β_6 and β_8 cases, the residual β_2 contribution can locally exceed the intended high-order contribution within a narrow interval around the phase-reference frequency.

In addition, we evaluated the relative importance of the different dispersion orders by comparing their dispersion lengths[5]. Because β_k in our notation represents the net intracavity dispersion accumulated over one cavity round trip, the dispersion-length expression can be written as

$$L_{D,n} = L_{\text{cav}} \frac{T_0^n}{|\beta_n|}, \quad (8)$$

where L_{cav} = 8.3 m and $T_0 = \Delta t_{\text{FWHM}}/1.763$. For β_k = -1.96 ps^k, the measured pulse durations of 1.62, 1.29, and 0.96 ps for β_4, β_6, and β_8 give characteristic high-order dispersion lengths of

$$L_{D,4} = 3.02 \text{ m}, \quad L_{D,6} = 0.65 \text{ m}, \quad L_{D,8} = 0.03 \text{ m}.$$

For the same respective pulse durations, the characteristic lengths associated with the maximum residual β_2 are 1.08 km, 0.68 km, and 0.38 km, while those associated with the maximum residual β_3 are 92.0 km,

46.5 km, and 19.1 km. This demonstrates that residual β_2 is not important in this system and that β_3 is also negligible.

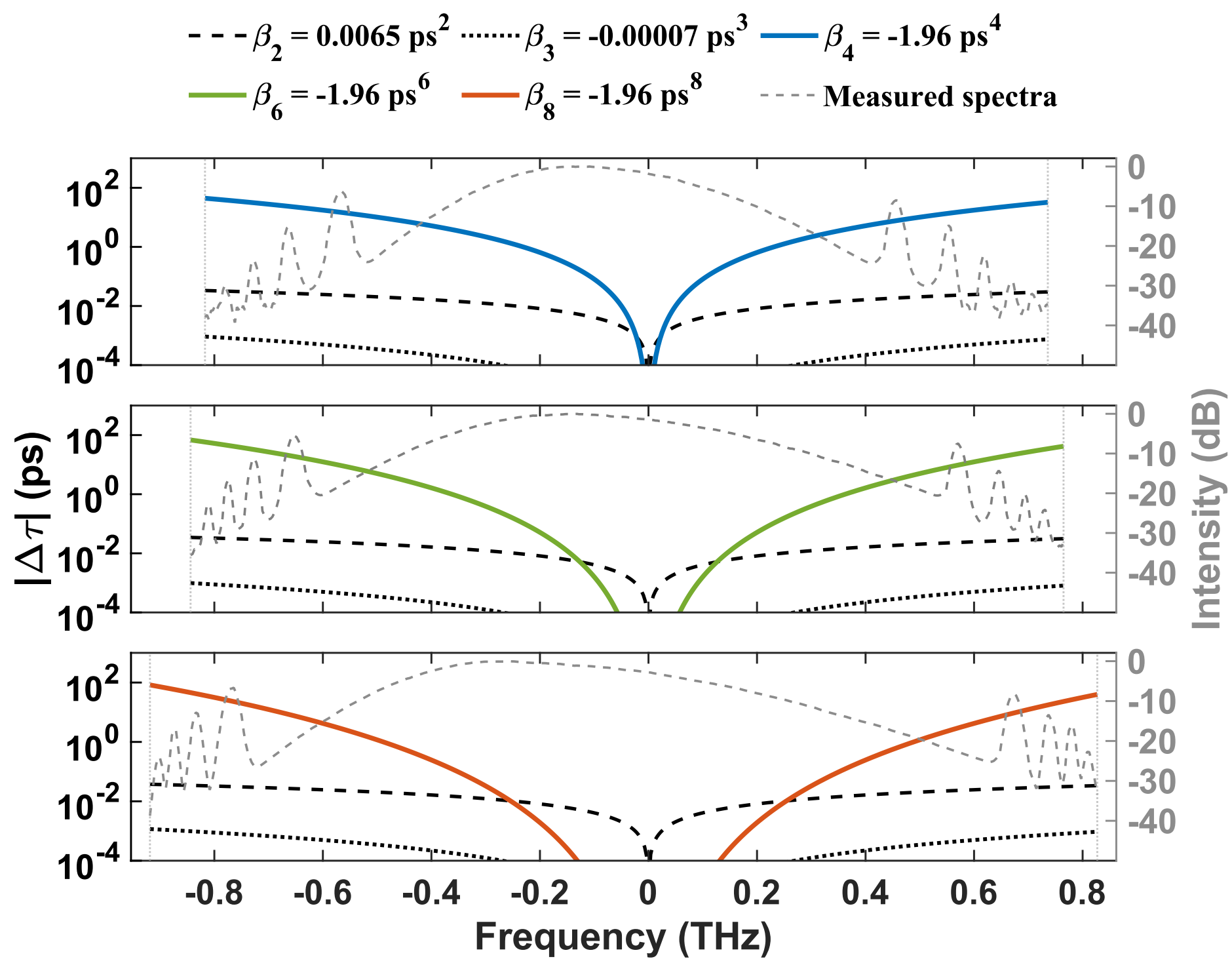

Supplementary Fig. 6 | Comparison of the group delay contributions from residual low-order and applied high-even-order dispersion. Frequency-dependent magnitudes of the group delay contributions for the β_4 **(a)**, β_6 **(b)**, and β_8 **(c)** PHEOD soliton states. The black dashed and dotted curves represent the contributions from the maximum residual intracavity dispersion values. The blue, green, and orange solid curves represent the applied high-even-order dispersion contributions. The gray dashed curves show the corresponding independently normalized experimental spectra plotted against the right-hand axis. The frequency offset is defined relative to the phase-reference frequency f_0, and the vertical dotted lines indicate the selected experimental spectral limits.

To further assess the influence of residual low-order dispersion, we performed a sensitivity analysis for the eighth-order case, which is expected to be the most sensitive to lower-order perturbations among the three PHEOD regimes considered here. In the simulations, we introduced controlled residual β_2 and β_3 terms and examined whether the characteristic linear dependence of $\Delta\omega^8$ on the sideband order m was preserved.

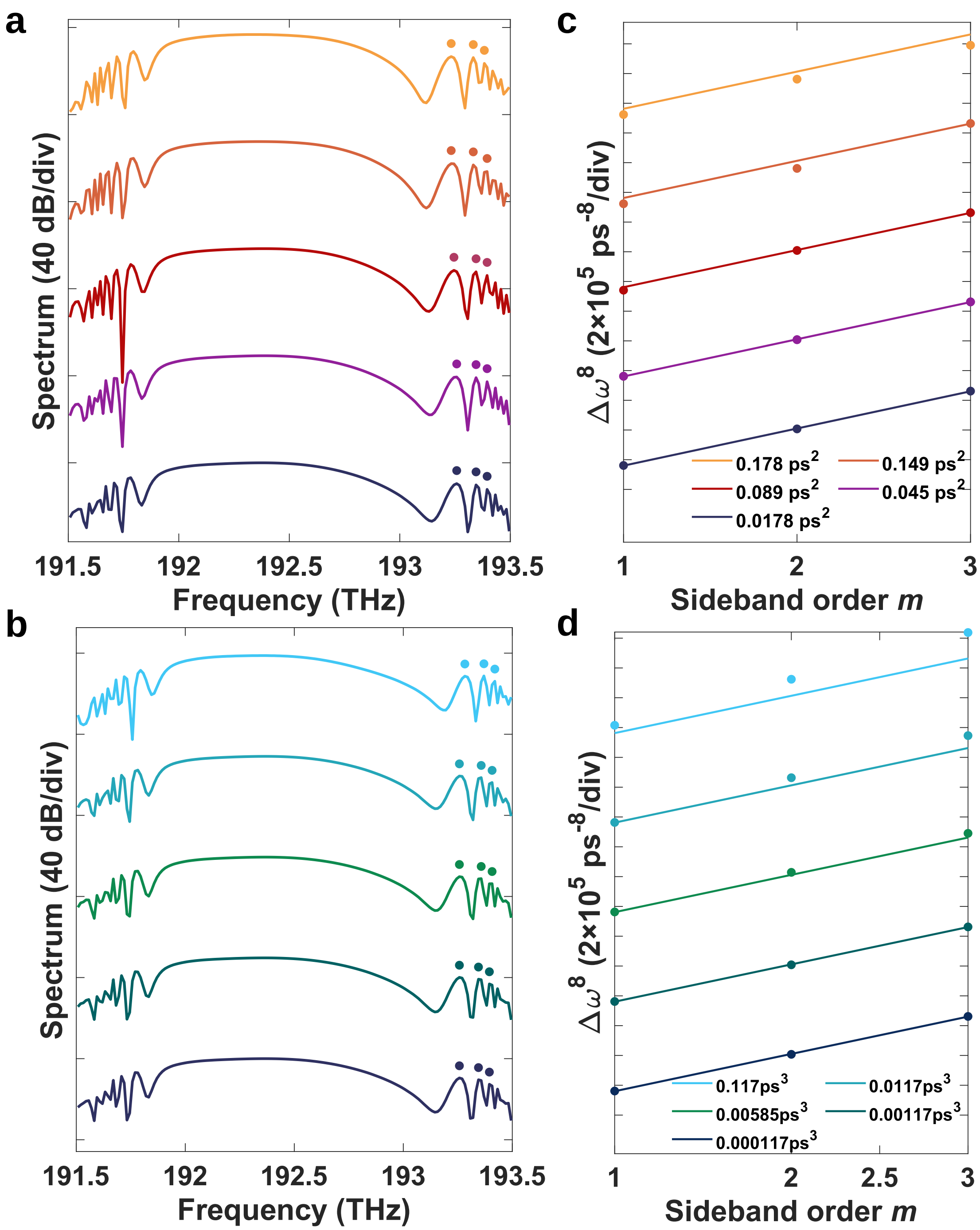

Supplementary Fig. 7 | Sensitivity analysis of spatiotemporally synchronized PHEOD solitons. a, b, Simulated spectra with different values of residual β_2 (**a**) and β_3 (**b**). The colored dots indicate the extracted Kelly sideband peaks. **c, d,** Corresponding quantitative analysis of sideband positions. Solid lines denote the theoretical linear fits.

As shown in Supplementary Fig. 7a,c, we introduced uncompensated β_2 ranging from 0.0178 ps^2 up to 0.178 ps^2 to identify the simulated tolerance limit. For residual β_2 values up to 0.089 ps^2, the defining linear relationship between $\Delta\omega^8$ and sideband order m is maintained. As the mismatch increases to 0.149 ps^2, a deviation from the theoretical fit begins to appear. When the mismatch reaches 0.178 ps^2, the data points deviate strongly from the linear fit. This identifies the approximate residual-β_2 level at which the eighth-order scaling is disrupted.

A similar analysis was performed for residual β_3. As shown in Supplementary Fig. 7b,d, the $\Delta\omega^8$ scaling is maintained for residual β_3 values up to 0.00585 ps^3. When β_3 is increased to 0.0117 ps^3, deviations from the linear fit begin to appear, and a much larger perturbation of 0.117 ps^3 leads to a substantial departure from the expected eighth-order scaling. These results show that the sideband-position analysis is sensitive to sufficiently large residual low-order dispersion, but remains robust within the residual-dispersion range relevant to our experiment.

This numerical analysis provides a quantitative tolerance estimate for the eighth-order case. The onset of visible deviation occurs at residual values of approximately β_2 = 0.149 ps^2 and β_3 = 0.0117 ps^3, while the scaling remains well preserved up to β_2 = 0.089 ps^2 and β_3= 0.00585 ps^3. In the experiment, the maximum residual values across the participating TMs are much smaller: β_2 = 0.0065 ps^2 and β_3 = -0.00007 ps^3. These values are more than one order of magnitude below the simulated perturbation levels that produce noticeable deviations.

Supplementary Note 5: PHEOD soliton formation in individual TMs with extended dispersion parameters

To confirm the universality of the TMDC technique for generating PHEOD solitons in MM fiber lasers, we extended our investigation beyond the dispersion parameters shown in Fig. 2 of the main text. Specifically, we performed a detailed analysis of PHEOD soliton generation and the corresponding spectral features across different TMs under both lower and higher dispersion magnitudes. The results indicate that all TMs maintain highly stable PHEOD soliton operation under regimes dominated by pure β_4, β_6, and β_8. Notably, as the magnitude of the introduced negative dispersion increases, we observe a clear narrowing of the spectral bandwidth across all TMs, confirming that the introduced high-order dispersion consistently dominates the pulse-shaping process. Overall, these experiments demonstrate that the system is not restricted to specific parameter values, but can generate different TM PHEOD solitons over a broad range of dispersion parameters.

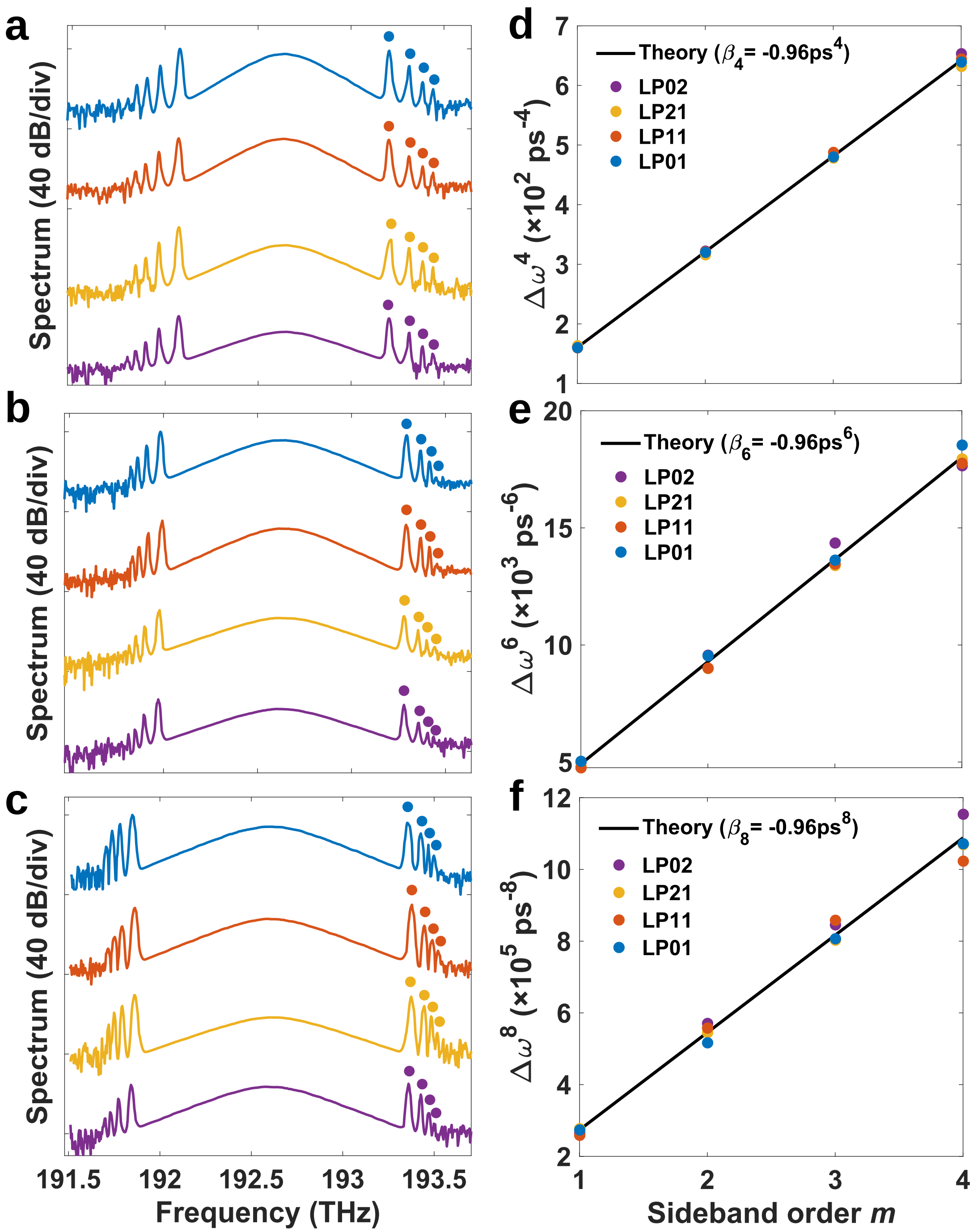

Supplementary Fig. 8 | Spectral characterization of PHEOD solitons in individual TMs under lower dispersion. Left column: Normalized experimental spectra recorded for four distinct TMs governed by pure β_4 (**a**), β_6 (**b**), and β_8 (**c**). The dominant dispersion coefficients were set to β_k = -0.96 ps^k. d–f, Corresponding analysis of Kelly sideband positions, illustrating the linear dependence of the *k*-th power of the $\Delta\omega^k$ on the *m*. Colored symbols denote distinct modes, while solid black lines indicate theoretical predictions.

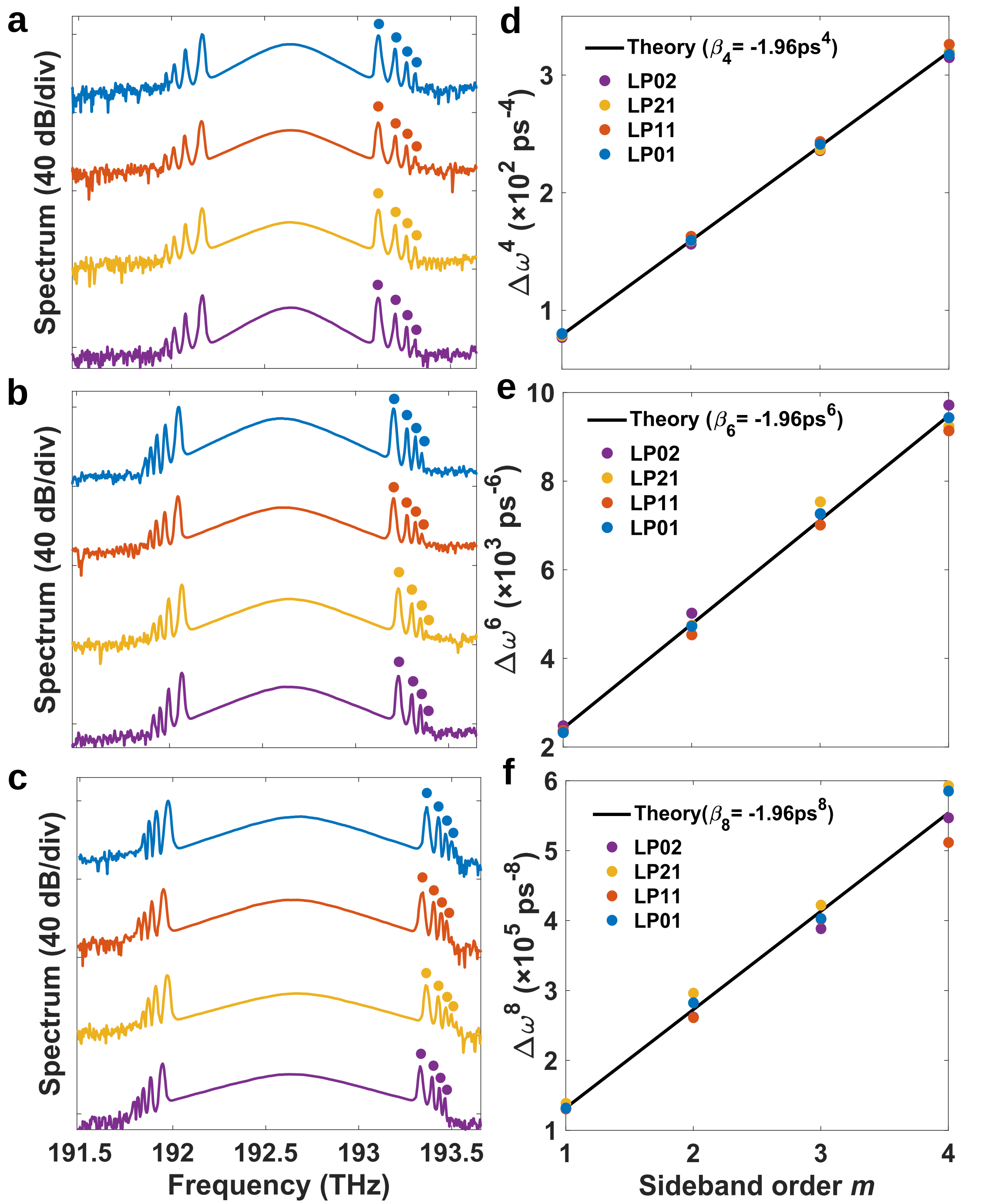

Supplementary Fig. 9 | Spectral characterization of PHEOD solitons in individual TMs under higher dispersion. Left column: Normalized experimental spectra recorded for four distinct TMs governed by pure β_4 (**a**), β_6 (**b**), and β_8 (**c**). The dominant dispersion coefficients were set to β_k = -1.96 ps^k. **d–f**, Corresponding analysis of Kelly sideband positions, illustrating the linear dependence of the *k*-th power of the $\Delta\omega^k$ on the *m*. Colored symbols denote distinct modes, while solid black lines indicate theoretical predictions.

Supplementary Note 6: Extraction of sideband energy via local baseline subtraction

The total output energy in the MM cavity contains both the central soliton component and resonant sidebands. To estimate the pulse energy excluding the resonant sidebands, we subtract the energy contained in the resolved sidebands from the total spectral energy. This is particularly important for the MM spectra, where the central spectral envelope can be asymmetric. We therefore use a local-baseline subtraction method to estimate the sideband contribution.

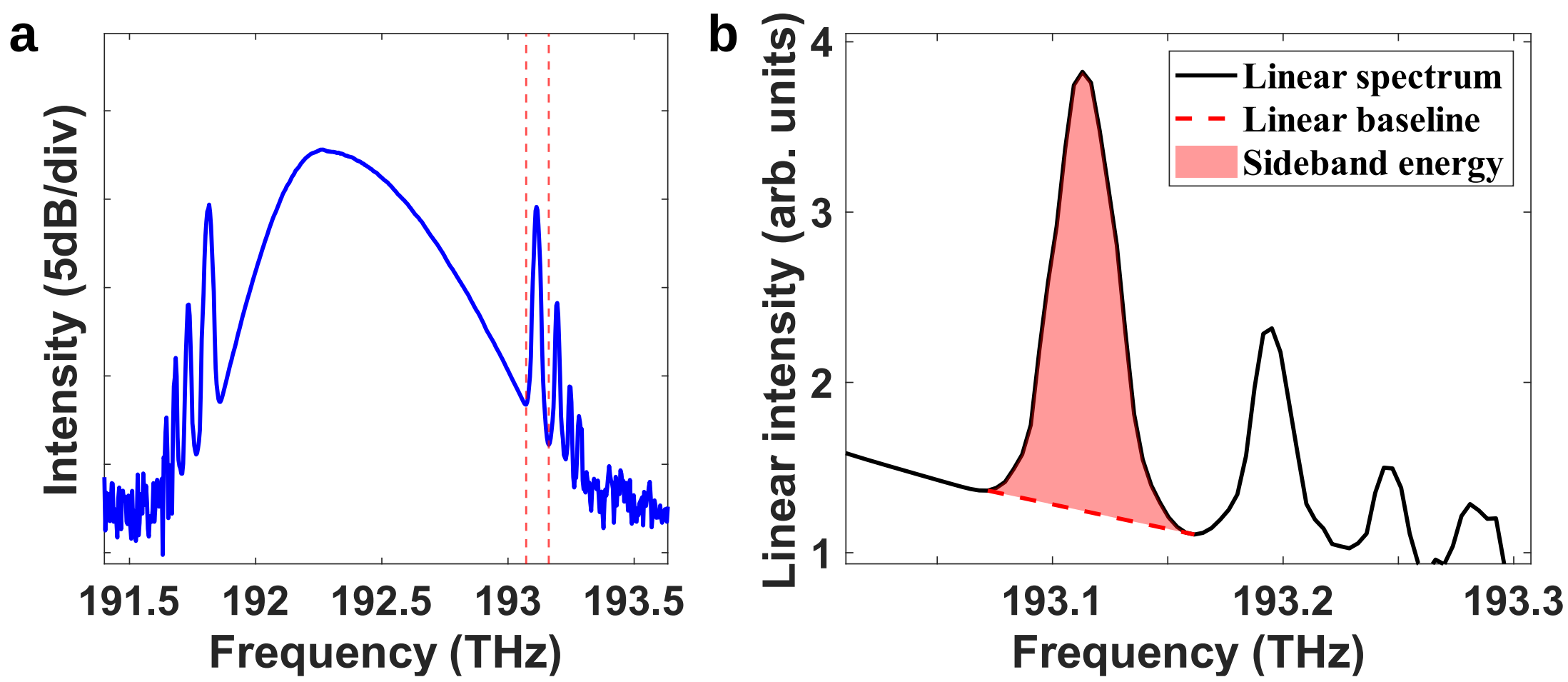

Supplementary Fig. 10 | Extraction of sideband energy. a, Original output spectrum plotted on a logarithmic scale. The red dashed lines mark the target sideband. **b**, Zoomed-in view of the target sideband on a linear scale, showing the local linear baseline (red dashed line) and the integrated sideband energy (red shaded area).

Although the experimental spectra are typically displayed on a logarithmic scale, such as dBm, to show high-dynamic-range spectral features (Supplementary Fig. 10a), all energy calculations are performed in the linear power domain (Supplementary Fig. 10b). For each resolved sideband on the high- and low-frequency sides, we define an integration window $[f_{\mathrm{left},\,i}, f_{\mathrm{right},\,i}]$. The two boundaries are chosen as the local spectral minima immediately adjacent to the corresponding sideband peak. Within this window, a local baseline is constructed by linear interpolation between the two boundary intensities (red dashed line in Supplementary Fig. 10b).

The sideband energy is then obtained by numerically integrating only the spectral power above this local baseline within the selected window. If no resolvable sideband peak is present, the local spectrum approaches the baseline and the extracted sideband contribution becomes correspondingly small. This procedure reduces over-subtraction of the central spectral component and provides a consistent estimate of the sideband energy across different dispersion orders and modal states.

Supplementary Note 7: Experimental initiation and evolution of mode-locking

As shown in Supplementary Fig. 11a, below the lasing threshold, the output spectrum is dominated by amplified spontaneous emission (ASE). As the pump power is increased, the laser enters the continuous-wave (CW) regime (Supplementary Fig. 11b), which is characterized by a narrow spectral peak and a speckled MM beam profile. To achieve the transition from CW operation to the mode-locked state, the intracavity PCs are adjusted to optimize nonlinear polarization rotation. When the pump power reaches the mode-locking threshold of 870 mW, the laser undergoes an abrupt transition into synchronized PHEOD soliton operation. This transition is accompanied by simultaneous changes in the temporal, spectral, and spatial properties of the output field. The narrow CW peak broadens into a wide, structured spectrum with resonant sidebands and the speckled CW beam evolves into a stable multimode beam profile, consistent with the formation of a synchronized multimode pulsed state.

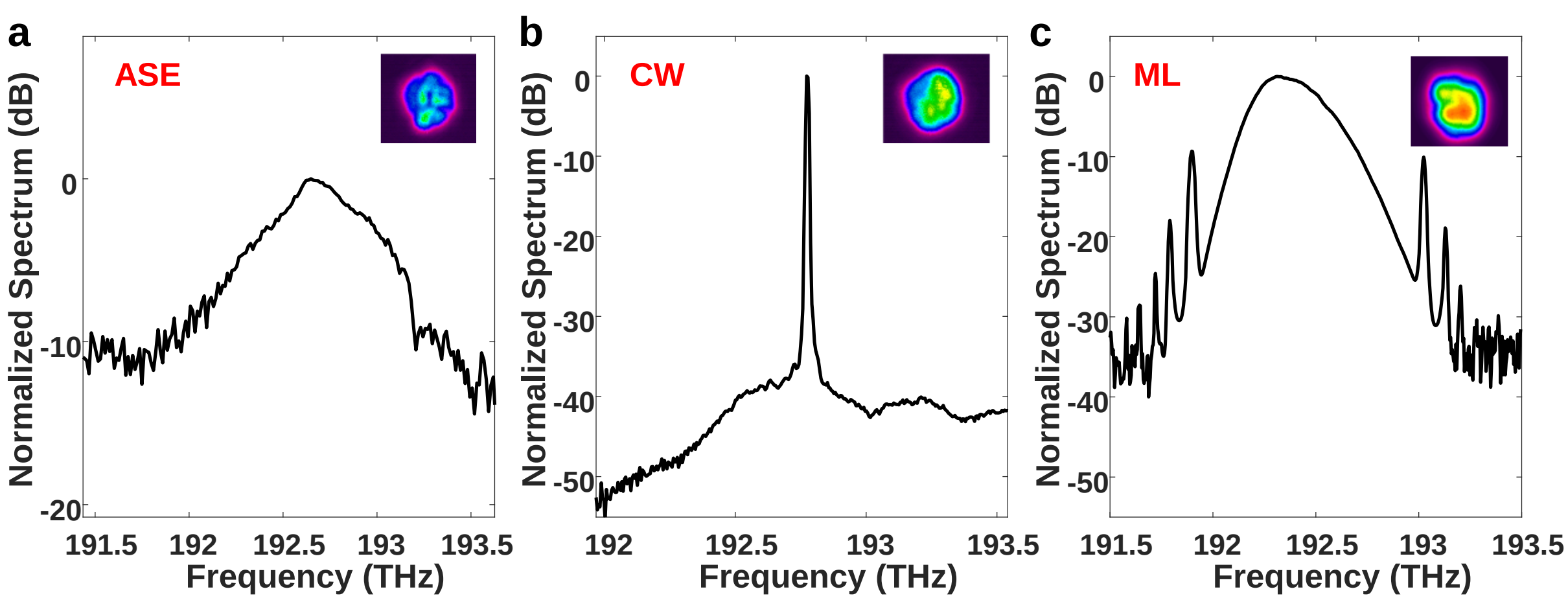

Supplementary Fig. 11 | Experimental observation of the transition to mode-locking. Normalized spectra and the corresponding near-field beam profiles (insets) for **a**, the amplified spontaneous emission (ASE) state, **b**, the continuous-wave (CW) lasing state, and **c**, the mode-locked state of synchronized PHEOD solitons.

Supplementary Note 8: Measurement of intermodal group velocity mismatch

To compensate the modal walk-off in the TMDC module, it is necessary to determine the GVM between the TMs supported by the FMF. We therefore used a time-of-flight (ToF) measurement[6] based on mode-selective excitation to characterize the differential mode group delay (DMGD) of the FMF used in the cavity.

The experimental setup is illustrated in Supplementary Fig. 12a. The output from a pulsed laser source was divided into two paths using a 1×2 optical splitter. One path served as the reference arm, where the pulse was coupled into the LP01 port of a mode MUX to excite the fundamental mode. The other path served as the test arm, connected sequentially to the LP11, LP21, or LP02 ports of the MUX to excite the target higher-order mode selectively. Variable attenuators (ATT 1 and ATT 2) were employed in each arm to balance the optical power. The combined modes were then launched into the fiber under test (FUT).

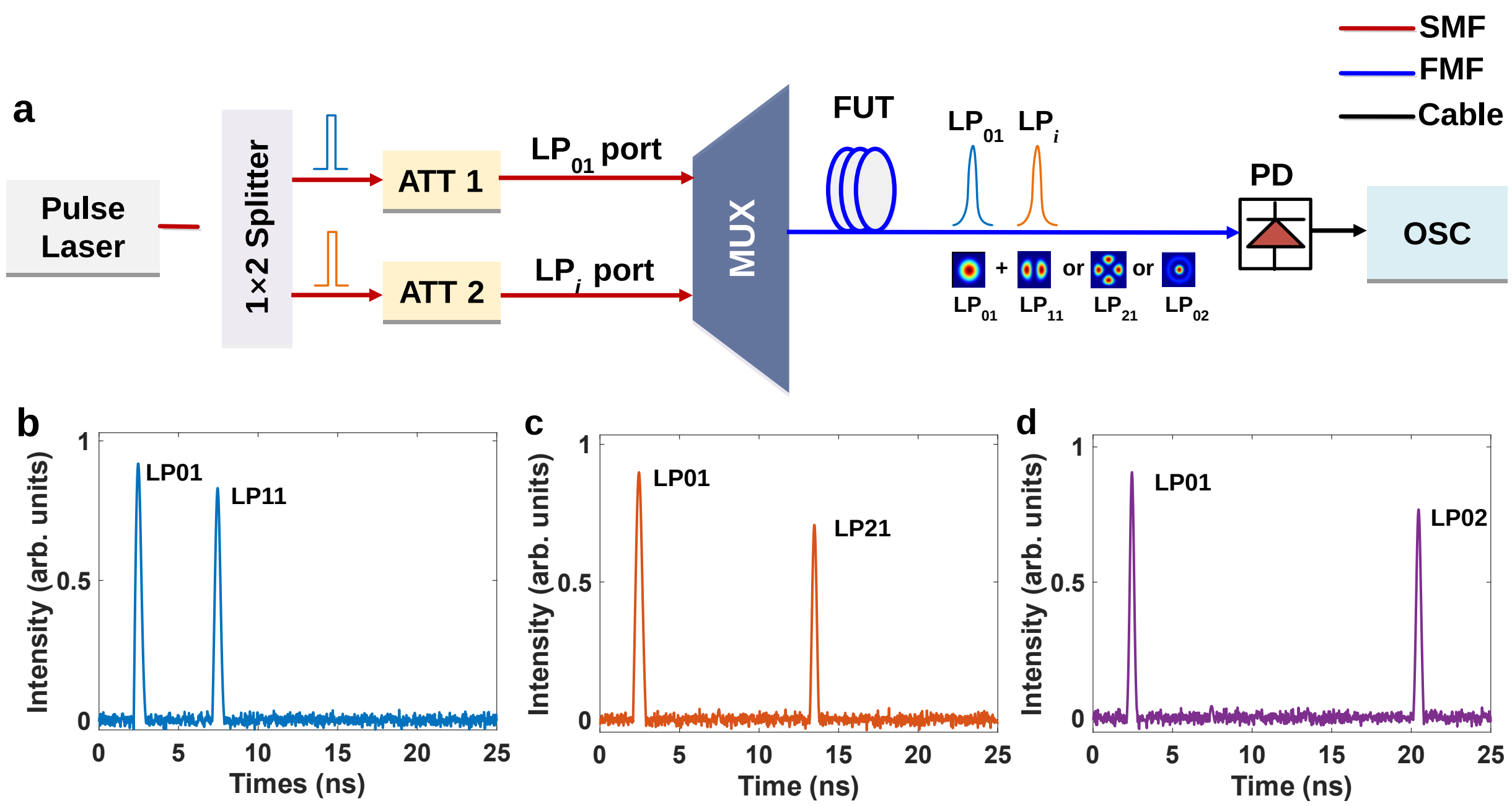

Supplementary Fig. 12 | Measurement of differential mode group delay based on the time-of-flight method. a, Schematic diagram of the experimental setup for intermodal dispersion measurement. A pulse laser is split into two paths to separately excite the fundamental mode (reference) and higher-order modes. ATT, attenuators; MUX, multiplexer; FUT, fiber under test; PD, photodetector; OSC, oscilloscope. **b–d**, Measured time-domain pulse waveforms showing the relative propagation delays between the LP01 and specific higher-order modes after propagating through the FUT: **(b)** LP01 and LP11, **(c)** LP01 and LP21, and **(d)** LP01 and LP02.

After propagation through the FUT, the different TMs accumulated different group delays. The output optical signals were converted into electrical signals by a photodetector and recorded with a digital oscilloscope. Supplementary Figs. 12b–d show the measured time-domain traces for LP01 together with LP11, LP21, and LP02, respectively. In each trace, the relative temporal separation between the LP01 peak and the higher-order-mode peak gives the intermodal group-delay difference. The DMGD between LP01 and mode j is calculated as:

$$DMGD_{1,j} = \Delta\tau_{1,j} / L = \left(t_1 - t_j\right) / L \quad (9)$$

where L is the length of the fiber under test; t_1 and t_j denote the relative propagation delays of the LP01 and the higher-order mode j, respectively; the subscripts 1, 2, 3, …, N correspond to LP01, LP11, LP21, LP02, etc. Based on the temporal separations observed in Supplementary Figs. 12b–d, the intermodal group delays relative to the LP01 mode were calculated to be 0.5 ps/m (LP11), 1.1 ps/m (LP21), and 1.8 ps/m (LP02), respectively. These experimentally measured values were used to determine the required compensation-fiber lengths in the TMDC module.

Supplementary Note 9: Mode-content controlled simulation

To demonstrate the impact of MM interactions on the pulse spectrum, we performed mode-content controlled numerical simulations under idealized dispersion conditions (β_2 = 0, β_3 = 0, β_6 = -1.44 ps^6). Supplementary Fig. 13 compares the simulated spectra for SM and four-mode operation. In the single-mode case, where only the fundamental mode is excited, the output spectrum is nearly symmetric and exhibits pronounced resonant sidebands (Supplementary Fig. 13a). The sideband-to-central energy ratio (SIR, defined as the ratio of the total energy in the left and right first-order sidebands to the energy in the central spectral region) is 26%.

When four TMs participate in the laser operation, the simulated state becomes spatiotemporally synchronized (Supplementary Fig. 13b). The solid curves show the spectra of the individual TM components, while the black dashed curve represents the total MM output spectrum. Compared with the SM case, the MM spectrum becomes asymmetric and the SIR decreases to 13%. This comparison shows that MM operation and intermodal coupling can contribute to the spectral asymmetry and sideband suppression observed experimentally, although the suppression is weaker in the simulations than in the measurements.

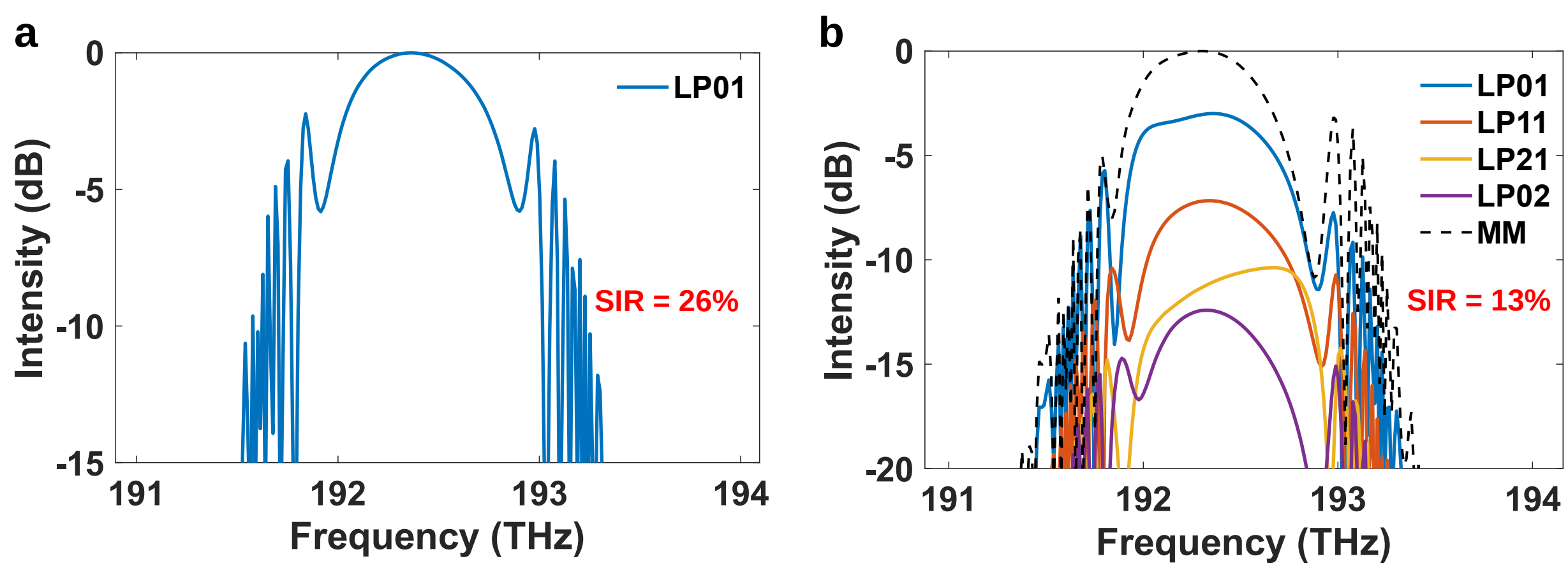

Supplementary Fig. 13 | Mode-content controlled numerical comparison of single-mode and multimode spectra. Simulated spectra for (**a**) SM operation and (**b**) four-mode operation under idealized dispersion conditions (β_2 = 0, β_3 = 0, and β_6 = -1.44 ps^6). In (**b**), the solid curves show the spectra of the constituent TMs, and the black dashed curve shows the total MM output spectrum.

Supplementary Note 10: Characterization of the MUX/DEMUX

The mode MUX/DEMUX employed in this system is based on an all-fiber cascaded fused biconical taper mode-selective coupler (MSC) structure. The operating principle relies on phase matching between the fundamental mode of a single-mode fiber and a target transverse mode of the few-mode fiber in the tapered coupling region, enabling selective conversion between the spatial channels. In the cascaded architecture, each MSC stage is designed to address a specific TM channel, while the full cascade performs mode multiplexing and demultiplexing for the four supported modes. The order of the MSC stages is chosen to reduce unwanted intermodal coupling and excess loss, rather than being an arbitrary sequence. The cascading logic is guided by supermode-propagation simulations, which track the modal energy evolution through the coupled-fiber structure[7].

To validate the practical performance of the MUX/DEMUX, we performed back-to-back characterization at an operating wavelength of 1550 nm. The measured insertion loss and intermodal crosstalk are summarized in Supplementary Table 2.

Supplementary Table 2 | The insertion loss and crosstalk of MUX/DEMUX (Unit: dB)

	LP01 OUT	LP02 OUT	LP11 OUT	LP21 OUT
LP01 IN	0.16	-36.80	-21.11	-29.13
LP02 IN	-34.47	0.38	-22.31	-31.55
LP11 IN	-20.33	-19.99	0.1	-19.03
LP21 IN	-28.19	-19.19	-20.59	0.34

Supplementary Note 11: Pulse-energy measurements in SM and synchronized MM operation

We measured the single-pulse energies of the synchronized MM states at the same pump power as the corresponding SM states. The measurements were performed for different dispersion orders (k = 4, 6, and 8) and different applied dispersion magnitudes. The SM data were obtained with only the LP01 channel oscillating, whereas the MM data correspond to synchronized four-TM operation.

As shown in Supplementary Fig. 14, the synchronized MM states generally exhibit higher pulse energies than the corresponding SM states under the same pump power. The dashed grey lines and labels indicate the measured MM-to-SM pulse-energy ratios under the same pump power. Depending on the dispersion order, dispersion magnitude, and pulse duration, the measured MM-to-SM pulse-energy ratios range from approximately 1.3 to 3.3.

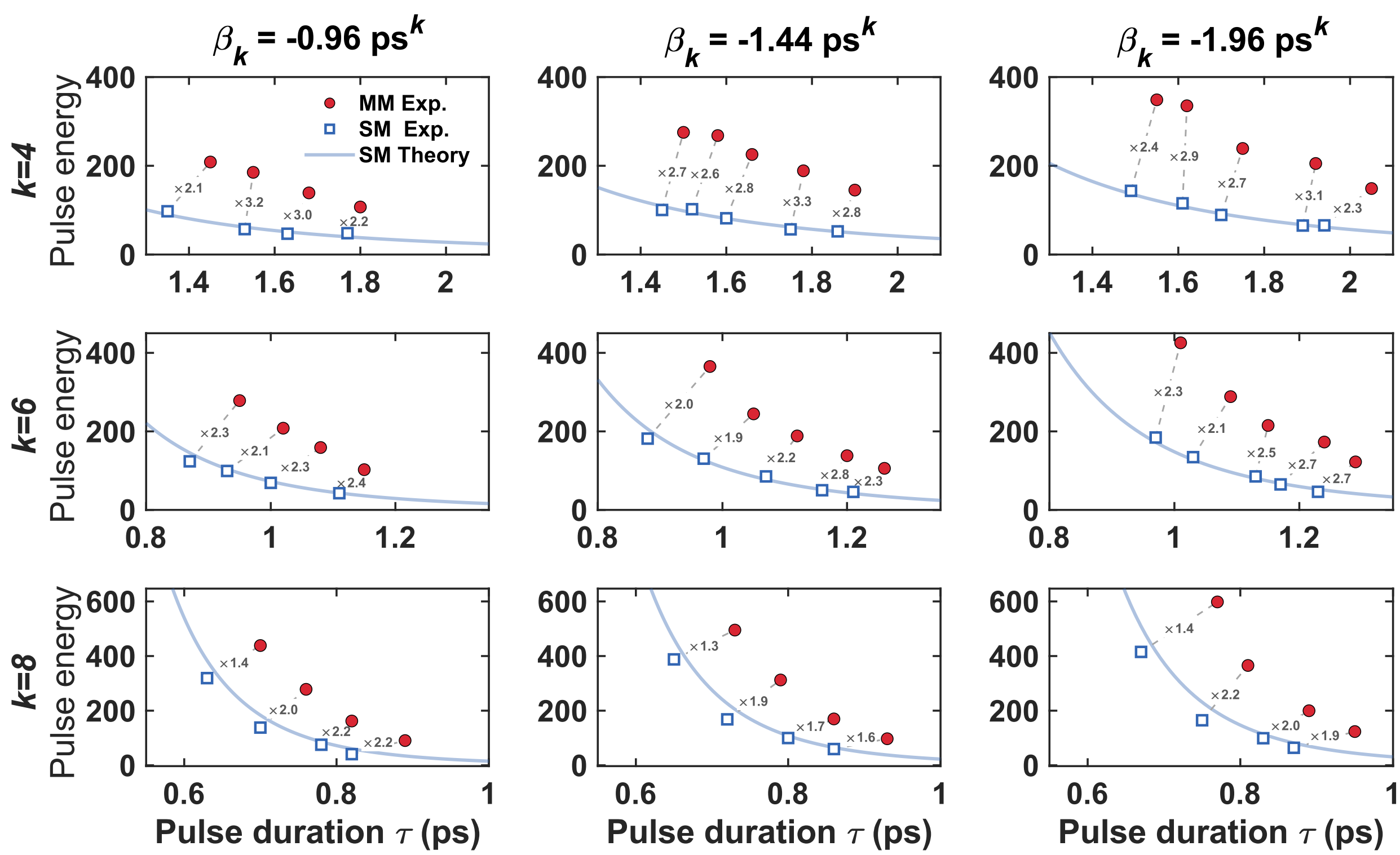

Supplementary Fig. 14 | Pulse-energy measurements in SM and synchronized MM operation under the same pump power. Measured pulse energy as a function of pulse duration for different dispersion orders k and applied dispersion magnitudes β_k. Red circles denote the synchronized MM states, blue open squares denote SM operation, and blue solid curves denote the theoretical energy-duration scaling of SM PHEOD solitons. Grey dashed lines connect corresponding SM and MM measurements obtained at the same pump power, and the labels indicate the measured MM-to-SM pulse energy ratios.

Supplementary References

1. Ito, T. et al. High-precision group-delay dispersion measurements of optical fibers via fingerprint-spectral wavelength-to-time mapping. *Photon. Res*. **4**, 13–16 (2016).
2. Liu, Y. et al. Pure-quartic solitons in multimode mode-locked fiber lasers. *Opt. Express* **33**, 30290–30303 (2025).
3. Tam, K. K. K., Alexander, T. J., Blanco-Redondo, A. & de Sterke, C. M. Stationary and dynamical properties of pure-quartic solitons. *Opt. Lett.* **44**, 3306-3309 (2019).
4. Runge, A. F. J. et al. Infinite hierarchy of solitons: Interaction of Kerr nonlinearity with even orders of dispersion. *Phys. Rev. Res.* **3**, 013166 (2021).
5. Blanco-Redondo, A. et al. Pure-quartic solitons. *Nat. Commun*. **7**, 10427 (2016).
6. Cheng, J. et al. Time-domain multimode dispersion measurement in a higher-order-mode fiber. *Opt. Lett.* **37**, 347–349 (2012).
7. Guo, H. et al. Ultra-low-loss all-fiber orbital angular momentum mode-division multiplexer based on cascaded fused-biconical mode selective couplers. *Adv. Photon. Nexus* **3**, 016006 (2024).
