# Supplementary material for: Spatiotemporally synchronized pure high-even-order dispersion solitons in multimode fiber lasers
Source: Nat Commun. 2026 Oct 3;17:11307. doi: 10.1038/s41467-026-78194-5 (PMC13724690; doi:10.1038/s41467-026-78194-5)
Supplement: Supplementary file 2 [file 41467_2026_78194_MOESM2_ESM.pdf]

nature portfolio

Peer Review File

Spatiotemporally synchronized pure high-even-order dispersion solitons in multimode fiber lasers

Corresponding Author: Professor Guijun Hu

This manuscript has been previously reviewed at another journal. This document only contains information relating to versions considered at Nature Communications.

This file contains all reviewer reports in order by version, followed by all author rebuttals in order by version.

Version 0:

Reviewer comments:

Reviewer #1

(Remarks to the Author)
This manuscript is a resubmission of the previous version. In my opinion, the authors have done an excellent job addressing the reviewers' comments, which has substantially improved the clarity of the results and strengthened the overall quality of the manuscript. I believe that, in its current form, the manuscript meets the high standards of Nature Communications, and I recommend it for publication.

Reviewer #2

(Remarks to the Author)
Following the first round of review, the authors have greatly improved the quality of the manuscript, in particular, the discussion related to the MM energy scaling. The new Supplemental Section related to the numerical simulation of intracavity modal walk-off and the synchronization mechanism is really insightful. Finally, other minor changes, such as incorporating the numerical simulation results directly into Fig. 4, greatly ease reading. Given these improvements and the quality of the experimental results, I recommend its publication in Nature Communications.

Reviewer #3

(Remarks to the Author)
This manuscript reports temporally synchronized high-even-order-dispersion soliton trains in a multimode mode-locked fiber laser using transverse-mode routing, modal-delay compensation, and spectral phase shaping. The topic is interesting and the experiment is technically nontrivial. However, the evidence supports a narrower conclusion than the manuscript's broader framing suggests. The work does not establish a fully coherent spatiotemporal soliton, a multimode energy-scaling platform, or truly independent per-mode dispersion engineering. This substantially limits the conceptual advance.

The novelty and motivation remain insufficiently defined. The manuscript should explicitly benchmark what is new relative to the closest prior multimode pure-quartic mode-locked fiber-laser work, rather than simply state that higher dispersion orders are included. In addition, the manuscript does not demonstrate a clear advantage over single-mode PHEOD lasers in pulse duration, pulse energy, or peak power. The added complexity of the multimode architecture is therefore not clearly justified.

The mode-control claim is also weaker than implied. The experiment applies a shared spectral phase profile to all separated channels, not independently optimized phase masks for each transverse mode. Thus, the work demonstrates mode routing and modal-delay compensation combined with a common high-even-order phase mask, but not full mode-specific chromatic-dispersion control.

The "pure" PHEOD claim is not fully established. Residual $\beta 2$ and $\beta 3$ remain, and mode-dependent deviations are visible, especially for $\beta 8$. Sideband scaling is a useful consistency check, but it is not sufficient to prove that the dynamics are governed by a single high-even-order term. A direct comparison of $\beta 2$, $\beta 3$, and βk phase or group-delay contributions over

the measured bandwidth is needed.

The evidence supports temporal synchronization, not intermodal phase coherence. RF repetition-rate locking and clean autocorrelation traces do not demonstrate phase locking or a coherent spatiotemporal soliton. Any wording implying a conventional coherent STML state should be removed unless phase-sensitive measurements are provided.

The proposed mechanisms and simulations remain mainly qualitative. Sideband suppression and spectral asymmetry are attributed to modal walk-off, intermodal nonlinear coupling, saturable absorption, residual dispersion, gain filtering, and cavity response, but no controlled experiment separates these contributions. The simulations reproduce some trends, but the key spectral features are not quantitatively matched, and sideband suppression is weaker in simulation than in experiment. These results should therefore be presented as qualitative support, not as established mechanistic proof.

Overall, the manuscript presents an interesting work, but the evidence does not yet support a broadly significant advance suitable for Nature Communications. Publication would require sharper novelty positioning, quantitative proof of high-even-order dominance, phase-sensitive evidence if coherence is implied, and controlled tests of the proposed multimode mechanisms.

Version 1:

Reviewer comments:

Reviewer #3

(Remarks to the Author)
The authors' response and additional analyses address several technical concerns. In particular, the new spectral-phase, group-delay, dispersion-length, and sensitivity analyses provide reasonable evidence that the applied high-even-order dispersion dominates over the residual β2 and β3. The authors have also clearly stated that they do not claim intermodal phase locking, coherent STML, or fully independent per-mode dispersion control. I therefore consider these specific issues adequately clarified.

Nevertheless, I remain unconvinced that the manuscript establishes a sufficiently broad and mechanistically resolved advance for Nature Communications. The authors correctly identify the novelty as the first experimental realization of synchronized multimode PHEOD states. However, pure-quartic solitons, PHEOD soliton lasers, and solitons governed by arbitrary negative even-order dispersion are already established [1–3]. Multimode synchronization and STML are also established frameworks [4,5], while multimode pure-quartic and PHEOD laser states have already been investigated theoretically [6,7]. The present advance is therefore primarily the experimental combination of these established elements, rather than a new soliton class, synchronization principle, or dispersion-engineering concept.

The authors emphasize that the multimode state self-organizes through nonlinear coupling. This defining interpretation remains insufficiently demonstrated. In the low-energy simulation, Kerr-mediated interactions and saturable-absorber gating are reduced simultaneously, so their individual roles cannot be distinguished. The single-mode/multimode comparison shows that multimode interactions can contribute to spectral asymmetry and sideband suppression, but the simulated suppression is weaker than in experiment. Moreover, this comparison assumes ideal β2 = β3 = 0, whereas the experiment uses a common phase mask and retains mode-dependent residual dispersion. These simulations establish plausibility, but not a uniquely identified or quantitatively validated synchronization mechanism.

This distinction is important because the TMDC already provides periodic linear compensation that prevents unbounded modal walk-off and restores temporal overlap once per round trip. The present results do not clearly establish whether the observed state is a distinct nonlinear multimode attractor or a synchronized cavity state primarily maintained by engineered delay compensation and common saturable-absorber gating. Independent numerical removal of XPM, other intermodal nonlinear terms, and saturable-absorber-mediated coupling—using the actual shared-mask residual dispersions—would be needed to support the stronger self-organization interpretation.

I accept the authors' clarification that performance optimization is not the objective, and I do not regard the absence of superior pulse energy or duration as a methodological flaw. However, once practical performance and energy scaling are excluded from the central claims, the impact must rest mainly on the discovery and understanding of a new physical state. Here, the state is explicitly not coherent STML, does not involve independent modal phase control, and its governing nonlinear mechanism remains unresolved.

Overall, the manuscript presents a technically sophisticated experimental implementation of synchronized multimode PHEOD pulse trains. However, the underlying PHEOD physics, multimode synchronization framework, and theoretical multimode PHEOD states are already established, while the present work does not demonstrate coherent STML, a new scaling law, independent modal functionality, or a uniquely resolved synchronization mechanism. I therefore remain unconvinced that the advance reaches the level of general significance expected for Nature Communications, although it would be suitable for a focused journal in ultrafast, nonlinear, or multimode photonics.

[1] Blanco-Redondo, A. et al. Pure-quartic solitons. Nature Communications 7, 10427 (2016).
[2] Runge, A. F. J. et al. The pure-quartic soliton laser. Nature Photonics 14, 492–497 (2020).
[3] Runge, A. F. J. et al. Infinite hierarchy of solitons: Interaction of Kerr nonlinearity with even orders of dispersion. Physical

Review Research 3, 013166 (2021).
[4] Wright, L. G., Christodoulides, D. N. & Wise, F. W. Spatiotemporal mode-locking in multimode fiber lasers. Science 358, 94–97 (2017).
[5] Wright, L. G. et al. Mechanisms of spatiotemporal mode-locking. Nature Physics 16, 565–570 (2020).
[6] Liu, Y. et al. Pure-quartic solitons in multimode mode-locked fiber lasers. Optics Express 33, 30290–30303 (2025).
[7] Liu, Y. et al. Pure high-even-order dispersion solitons in multimode mode-locked fiber lasers. Chaos, Solitons & Fractals 207, 118014 (2026).

Open Access This Peer Review File is licensed under a Creative Commons Attribution 4.0 International License, which permits use, sharing, adaptation, distribution and reproduction in any medium or format, as long as you give appropriate credit to the original author(s) and the source, provide a link to the Creative Commons license, and indicate if changes were made.
In cases where reviewers are anonymous, credit should be given to 'Anonymous Referee' and the source.
The images or other third party material in this Peer Review File are included in the article’s Creative Commons license, unless indicated otherwise in a credit line to the material. If material is not included in the article’s Creative Commons license and your intended use is not permitted by statutory regulation or exceeds the permitted use, you will need to obtain permission directly from the copyright holder.
To view a copy of this license, visit https://creativecommons.org/licenses/by/4.0/

Reviewer #1 (Remarks to the Author):

This manuscript is a resubmission of the previous version. In my opinion, the authors have done an excellent job addressing the reviewers' comments, which has substantially improved the clarity of the results and strengthened the overall quality of the manuscript. I believe that, in its current form, the manuscript meets the high standards of Nature Communications, and I recommend it for publication.

Reviewer #2 (Remarks to the Author):

Following the first round of review, the authors have greatly improved the quality of the manuscript, in particular, the discussion related to the MM energy scaling. The new Supplemental Section related to the numerical simulation of intracavity modal walk-off and the synchronization mechanism is really insightful. Finally, other minor changes, such as incorporating the numerical simulation results directly into Fig. 4, greatly ease reading. Given these improvements and the quality of the experimental results, I recommend its publication in Nature Communications.

Response: It is gratifying that these reviewers find the manuscript to be substantially improved, and that it reaches the high standards of *Nature Communications*.

Reviewer #3 (Remarks to the Author):

Comment (1): This manuscript reports temporally synchronized high-even-order-dispersion soliton trains in a multimode mode-locked fiber laser using transverse-mode routing, modal-delay compensation, and spectral phase shaping. The topic is interesting and the experiment is technically nontrivial. However, the evidence supports a narrower conclusion than the manuscript's broader framing suggests. The work does not establish a fully coherent spatiotemporal soliton, a multimode energy-scaling platform, or truly independent per-mode dispersion engineering. This substantially limits the conceptual advance.

Response (1): We thank the reviewer for this careful assessment and for the opportunity to clarify the scope of our claims. We wish to point out that the paper never claims either generation of a fully coherent spatiotemporal soliton nor observation of spatiotemporal mode-locking (STML). The main claim of the paper is spatiotemporal synchronization of pulses in the different modes. The introduction mentions, and the discussion section addresses the close relationship of the present work with STML. The text states that future studies may address STML in a similar setting.

The title, Abstract, and Introduction do not claim the observation of STML or the generation of coherent spatiotemporal solitons. Nevertheless, we appreciate the reviewer's concern that the broader framing could give this impression, and we have revised the wording in several places to make the advances of the present work, as well as what is not claimed, explicit. In short, we do not claim coherent pulse generation or STML because we do not prove phase-locking, and the limited temporal overlap of the pulses as they traverse the cavity precludes labeling of the operation as STML in our opinion. On the other hand, as we discuss, the

observed operation shares some essential properties with STML: the multimode (MM) states are the result of self-organization of the longitudinal and transverse modes (TMs) through nonlinear coupling, the synchronization of the modes is quantitatively close to that observed in STML lasers, and based on available measurements the pulses are evidently close to transform-limited.

In addition, we agree that the phrase “truly independent per-mode dispersion engineering” may overstate the practical capability of the TMDC. In fact, we have already revised this statement in the manuscript to avoid implying perfectly independent control of each transverse mode. Importantly, the sensitivity analysis shows that we do not expect the imperfections of the TMDC to seriously hinder the desired laser operations. And the real evidence is in the experimental results – the generation of the PHEOD solitons in each mode is clear. In any case, we have stated this point more clearly in the revised manuscript and taken care not to overclaim the capabilities of the TMDC.

Corresponding modifications in the revised manuscript:

To address the above concerns and avoid any potential overstatement, we have revised the Abstract, Introduction, Results, and Discussion sections as follows:

(Line 20) We have added: *"These multimode pulsed states self-organize through nonlinear coupling among the transverse modes."*

(Line 81) We have revised: *" In MM operation, synchronized trains of solitons in individual modes self-organize as a result of intermodal nonlinear interactions in the laser. Numerical simulations agree with the experimental results and provide insight into the behavior and properties of the laser. Pulses in individual modes overlap temporally only in a portion of the cavity, so these new MM pulsed lasing states do not represent conventional STML. However, the laser exhibits some of the signatures of STML, and the relationship of the spatiotemporal wave propagation in the laser to STML will be discussed."*

(Line 190) We have revised: *"Despite the different mode field distributions of the various TMs, the variation of the spectra with dispersion order is quite consistent even with the small imperfections in dispersion control."*

(Line 257) We have revised: *"Nonlinear interactions are required to synchronize the fields in different TMs."*

(Line 300) We have revised: *"The TMDC technique facilitates this by spatially decoupling the TMs before applying phase shaping. Given that pulses in different modes do not completely overlap temporally, we would not describe the operation of the presented laser as STML, nor do we claim true phase-locking of the distinct TMs. Nevertheless, the results presented here represent a major step toward STML in PHEOD systems."*

(Line 318) We have deleted: *"We believe that this laser is a spatiotemporal version of coupled cavities, in which different modes or pulses interact through a common element or process, but remain independent owing to their temporal separation."*

Comment (2): The novelty and motivation remain insufficiently defined. The manuscript should explicitly benchmark what is new relative to the closest prior multimode pure-quartic mode-locked fiber-laser work, rather than simply state that higher dispersion orders are included.

Response (2): We thank the reviewer for raising this important point. The closest prior work on MM pure-quartic solitons was based on numerical simulations and provided a theoretical investigation of their dynamics[R1-R3]. There is no prior experimental study of lasers with multiple TMs and any higher-order dispersion. Specifically, there is no prior experimental study of a laser with multiple TMs and pure-quartic dispersion. Studies with higher dispersion orders are a secondary point relative to the observation of MM synchronized states at any given dispersion order.

Importantly, these synchronized MM states are not simply a superposition of independently generated solitons in different TMs. They emerge through nonlinear coupling among the participating TMs and exhibit temporal, spectral, and spatial properties that differ from the corresponding individual-TM states. Thus, the experiment reveals new MM pulsed states of a laser.

We show that in MM operation, trains of solitons in individual modes can be synchronized through spatiotemporal nonlinear interactions in the laser. The resulting MM states emerge from nonlinear coupling of the distinct TMs. These properties are quite similar to those expected in STML. However, we do not believe that a coherent MM wave packet traverses the entire laser cavity. As a result, phase-locking of different TMs may or may not occur. We do not study this experimentally. Considering these points, we do not claim to observe STML in this work. We refer to spatiotemporal synchronization of the pulses in distinct modes. We do discuss the relationship between the pulse states observed here with STML.

Corresponding modifications in the revised manuscript:

To clarify the novelty of the present work, we have revised the Abstract and Introduction to explicitly emphasize the first experimental realization of synchronized PHEOD solitons in a multimode fiber laser:

(Line 15) We have revised: *"Here, we report the first experimental generation of synchronized PHEOD solitons in a multimode fiber laser."*

(Line 76) We have revised: *"Here, we report the first experimental generation of synchronized PHEOD solitons in an MM fiber laser."*

(Lines 84) The text is revised to emphasize that this work reports the observation of new pulsed states of a laser: *"Pulses in individual modes overlap temporally only in a portion of the cavity, so these new MM pulsed lasing states do not represent conventional STML."*

Comment (3): In addition, the manuscript does not demonstrate a clear advantage over single-mode PHEOD lasers in pulse duration, pulse energy, or peak power. The added complexity of the multimode architecture is therefore not clearly justified.

Response (3): We thank the reviewer for pointing this out. The aim of this work is the experimental realization and systematic scientific investigation of synchronized multimode PHEOD states, rather than a demonstration of superior laser performance. No attempt was made to optimize the performance of the laser. We do not intend to present MM operation as offering performance advantages over single-mode (SM) PHEOD lasers. There is no clear way to compare the performance of a laser with an MM beam to one with a single-mode (SM) beam. Future work may address that along with ways to control the beam quality in an MM laser. We present data on the MM pulse energies in the Supplement as part of the scientific documentation of the pulsed laser states that are observed in this study. We think it is reasonable to mention potential energy scaling as a motivation, in the Introduction to the paper.

In the bigger picture, any discussion of performance of PHEOD lasers should include all existing lasers if it is intended to be a comparison of potentially-practical short-pulse sources. As the reviewer suggests, the complexity of any PHEOD laser will be high, and it will be difficult to justify given that modern femtosecond lasers built with commercial off-the-shelf components can reach pulse energies about 100 times higher than those of a PHEOD soliton laser at a fraction of the complexity and cost of the PHEOD laser.

Corresponding modifications in the revised manuscript:

(1) Regarding the potential for energy scaling, we have slightly revised a sentence there to say: **(Line 73)** "*Experimental investigations of PHEOD soliton formation in MM fiber lasers will be fruitful scientifically, and it may be possible to scale the stable pulse energy by leveraging the combination of MM and PHEOD properties.*"

(2) We have revised the statement associated with Fig. S12 (now Fig. S13) to report the measured pulse energies only as experimental characterization, without implying practical energy scaling:
(Line 236) *"Additional measurements under the same pump power are provided in Fig. S13, which further characterize the pulse energies of the synchronized MM states."*

(3) In addition, we have substantially revised Supplementary Note 11 to present the pulse-energy measurements as experimental characterization of the SM and synchronized MM states, without making a claim of practical energy scaling.

Comment (4): The mode-control claim is also weaker than implied. The experiment applies a shared spectral phase profile to all separated channels, not independently optimized phase masks for each transverse mode. Thus, the work demonstrates mode routing and modal-delay compensation combined with a common high-even-order phase mask, but not full mode-specific chromatic-dispersion control.

Response (4): We thank the reviewer for this comment. We note that the submitted manuscript already described the limitations of the present implementation and did not intend to claim fully independent chromatic-dispersion control of each TM. In the Results section (Lines 123–126), we stated that, due to pulse-shaper constraints, a unified phase profile was applied across the distinct TM channels. In addition, this limitation was explicitly discussed in the Discussion section (Lines 321–323), where we stated that the current pulse-shaper hardware applies a globally uniform dispersion phase profile to all TMs and therefore cannot precisely compensate for the residual dispersion differences between them. Nevertheless, we appreciate that some wording may have suggested stronger mode-specific dispersion control than intended. We have therefore revised the relevant text to make this limitation clearer.

Importantly, our sensitivity analysis indicates that these imperfections are not expected to significantly hinder the intended laser operation. More directly, the experimental results clearly demonstrate the generation of PHEOD solitons in each TM.

Corresponding modification in the revised manuscript:

To avoid any possible ambiguity, we have revised the corresponding wording in the manuscript:

(Line 78) *"...ii) control of the chromatic dispersion in the TM channels."*

(Line 127) "*...applying the same phase profile to each TM channel to imprint strong, negative high-even-order dispersion...*"

(Line 190) *"Despite the different mode field distributions of the various TMs, the variation of the spectra with dispersion order is quite consistent even with the small imperfections in dispersion control."*

Comment (5): The "pure" PHEOD claim is not fully established. Residual β_2 and β_3 remain, and mode-dependent deviations are visible, especially for β_8. Sideband scaling is a useful consistency check, but it is not sufficient to prove that the dynamics are governed by a single high-even-order term. A direct comparison of β_2, β_3, and β_k phase or group-delay contributions over the measured bandwidth is needed.

Response (5): We thank the reviewer for this important comment. We agree that the sideband-scaling relation primarily provides a consistency check and is not sufficient to establish that the pulse dynamics are dominated by the intended high-even-order dispersion term.

In Supplementary Note 4 of the submitted manuscript, we had already compared the frequency-dependent spectral-phase contributions of the residual β_2 and β_3 with those of the applied β_4, β_6, and β_8. We have now further added a comparison of the corresponding group-delay contributions and overlaid the calculated results with the measured spectra.

The dispersive spectral phase can be written as:

$$\Phi(\Omega) = \sum_{k\geq 2} \frac{\beta_k}{k!}\Omega^k. \tag{R1}$$

The group-delay relative to the central frequency is obtained by differentiation:

$$\Delta\tau(\Omega) = \frac{d\Phi}{d\Omega} = \sum_{k\geq 2} \frac{\beta_k}{(k-1)!}\Omega^{k-1}. \tag{R2}$$

Therefore, the contribution from an individual *n*-th-order dispersion term is

$$\Delta\tau_k(\Delta f) = \frac{\beta_k}{(k-1)!}(2\pi\Delta f)^{k-1}. \tag{R3}$$

where $\Omega = 2\pi\Delta f$ and $\Delta f = f - f_0$. Using this expression, we plotted the group delay contributions over the experimentally measured spectral for the β_4, β_6, and β_8, as shown in the new Fig. S5. The largest residual low-order dispersion values measured among the participating TMs, β_2 = 0.0065 ps^2 and β_3 = -0.00007 ps^3, are compared with the group delay contributions of the applied β_4, β_6, and β_8, for which β_k = -1.96 ps^k (k=4,6, and 8). The corresponding experimental spectra are independently normalized and overlaid to indicate the actual frequency ranges occupied by the central spectra, spectral wings, and resolved resonant sidebands.

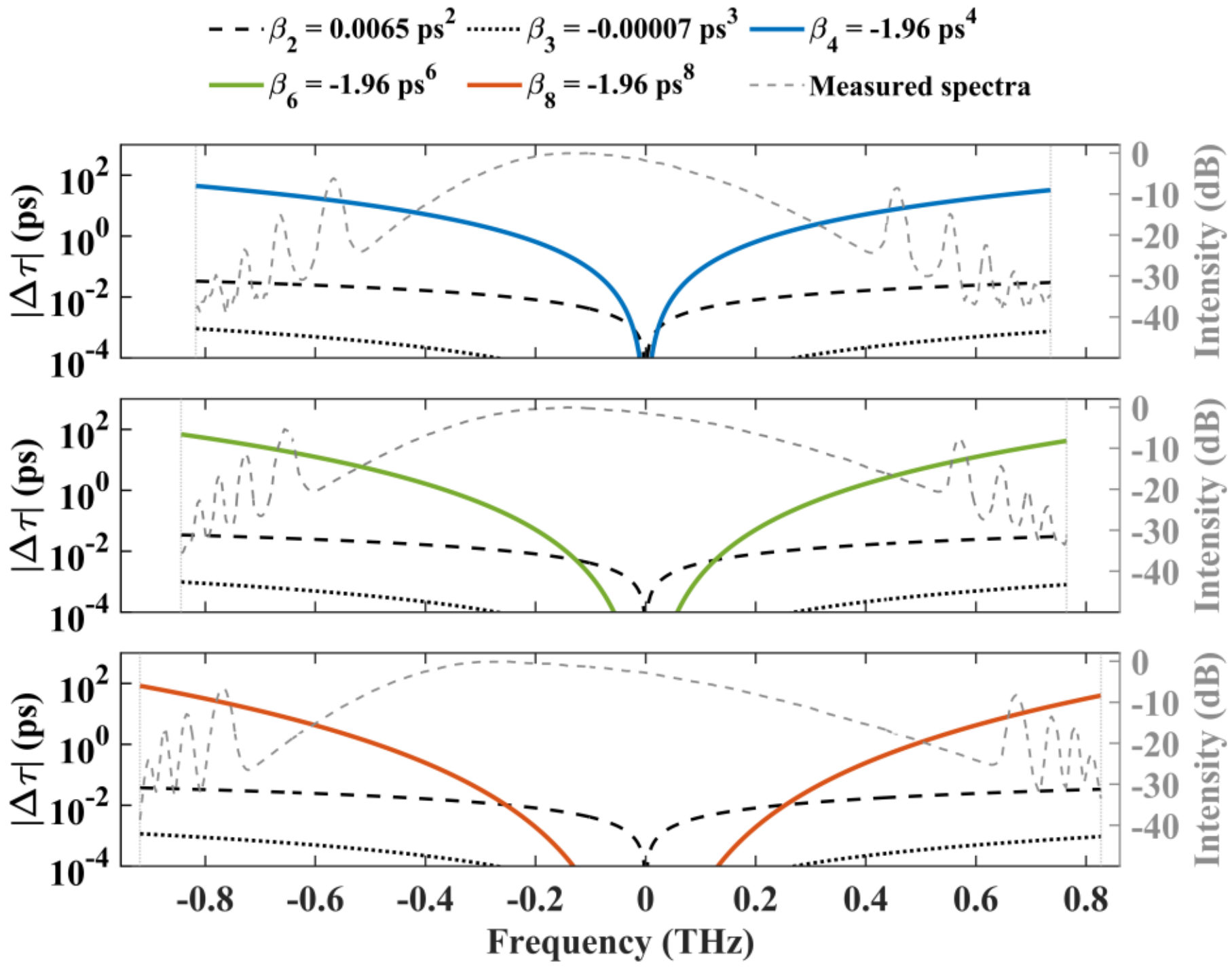

Fig. R1 Comparison of the group delay contributions from residual low-order and applied high-even-order dispersion. Frequency-dependent magnitudes of the group delay contributions for the β_4 **(a)**, β_6 **(b)**, and β_8 **(c)** PHEOD soliton states. The black dashed and dotted curves represent the contributions from the maximum residual intracavity dispersion values. The blue, green, and orange solid curves represent the applied high-even-order dispersion contributions. The gray dashed curves show the corresponding independently normalized experimental spectra plotted against the right-hand axis. The frequency offset is

defined relative to the phase-reference frequency f_0, and the vertical dotted lines indicate the selected experimental spectral limits.

As shown in Fig. R1, the applied high-even-order group delay contributions increase much more rapidly with frequency offset than the residual low-order contributions. For the β_4, β_6, and β_8, the intended high-even-order terms dominate in the experimentally observed spectral-wing and resolved resonant-sideband regions, where the characteristic PHEOD spectral features occur. In the β_6 and β_8 cases, the residual β_2 contribution can locally exceed the intended high-order contribution within a narrow interval around the phase-reference frequency.

In addition, following the dispersion length criterion used in the previously reported pure-quartic-soliton study[R4], we evaluated the relative importance of the different dispersion orders by comparing their characteristic dispersion lengths. Because β_k in our notation represents the net intracavity dispersion accumulated over one cavity round trip, the dispersion-length expression can be written as

$$L_{D,n} = L_{\mathrm{cav}} \frac{T_0^n}{|\beta_n|}, \tag{R4}$$

where $L_{\mathrm{cav}} = 8.3$ m and $T_0 = \Delta t_{\mathrm{FWHM}}/1.763$. For $\beta_k = -1.96$ ps^k, the measured pulse durations of 1.62, 1.29, and 0.96 ps for β_4, β_6, and β_8 give characteristic high-order dispersion lengths of

$$L_{D,4} = 3.02 \text{ m}, \quad L_{D,6} = 0.65 \text{ m}, \quad L_{D,8} = 0.03 \text{ m}.$$

For the same respective pulse durations, the characteristic lengths associated with the maximum residual β_2 are 1.08 km, 0.68 km, and 0.38 km, while those associated with the maximum residual β_3 are 92.0 km, 46.5 km, and 19.1 km. This demonstrates that residual β_2 is not important in this system and that β_3 is also negligible.

Taken together, the spectral-phase comparison already included in the originally submitted manuscript, the newly added group delay and dispersion length analyses, and the agreement between experiment and numerical simulation collectively indicate that the residual β_2 and β_3 are not important in this system. The applied high-even-order dispersion remains the dispersive contribution governing the observed soliton states. Here, "pure" does not imply that the residual low-order dispersion is zero, but rather that the intended high-even-order dispersion dominates the pulse-formation dynamics.

Corresponding modifications in the revised manuscript:

We have revised the relevant statements in the main text to avoid implying that the residual low-order dispersion is exactly zero. In addition, we have expanded Supplementary Note 4 to provide quantitative comparisons of both the group-delay contributions and the dispersion lengths.

(1) **(Line 138)** We have revised: *"Although this approach introduces minor dispersion mismatches among the different TMs, quantitative comparisons of the spectral-phase and group-delay contributions, together with a sensitivity analysis, show that the residual terms do not compromise PHEOD soliton formation (see Supplementary Note 4)."*

(2) Supplementary Note 4 has been expanded to include Eqs. (S6)–(S8) and Fig. S5, providing quantitative comparisons of the group-delay contributions and dispersion lengths associated with the residual low-order and applied high-even-order dispersion terms.

Comment (6): The evidence supports temporal synchronization, not intermodal phase coherence. RF repetition-rate locking and clean autocorrelation traces do not demonstrate phase locking or a coherent spatiotemporal soliton. Any wording implying a conventional coherent STML state should be removed unless phase-sensitive measurements are provided.

Response (6): We agree completely with these statements from the reviewer. The manuscript never states that we observe STML, and we did not intend to suggest it. In the revised version, we have added text to state explicitly and explain that we do not claim phase-locking, and thus do not claim STML.

Corresponding modification in the revised manuscript:

We have added text to clarify that we do not claim STML or intermodal phase locking:

(Line 84) *"Pulses in individual modes overlap temporally only in a portion of the cavity, so the observed MM states do not represent conventional STML. However, the laser exhibits some phenomena that occur in STML, and the relationship of the spatiotemporal wave propagation in the laser to STML will be discussed."*

(Line 300) *"Given that pulses in different modes do not completely overlap temporally, we would not describe the operation of the presented laser as STML, nor do we claim true phase-locking of the distinct transverse modes."*

Comment (7): The proposed mechanisms and simulations remain mainly qualitative. Sideband suppression and spectral asymmetry are attributed to modal walk-off, intermodal nonlinear coupling, saturable absorption, residual dispersion, gain filtering, and cavity response, but no controlled experiment separates these contributions. The simulations reproduce some trends, but the key spectral features are not quantitatively matched, and sideband suppression is weaker in simulation than in experiment. These results should therefore be presented as qualitative support, not as established mechanistic proof.

Response (7): We thank the reviewer for this comment. We note that the submitted manuscript does not collectively attribute the observed spectral asymmetry and sideband suppression to all of the mechanisms listed by the reviewer. In the current version, these spectral changes are discussed more cautiously, and Supplementary Note 9 states only that MM operation and intermodal coupling can contribute to the experimentally observed features.

The simulations themselves are quantitative, including the actual parameters of the experimental system. The reviewer is correct that it is difficult to isolate effects with experimental proof. Sideband suppression is treated primarily in Supplementary Note 9, where the text states, “Compared with the SM case, the MM spectrum becomes asymmetric and the

SIR decreases to 13%. This comparison shows that MM operation and intermodal coupling can contribute to the spectral asymmetry and sideband suppression observed experimentally, although the suppression is weaker in the simulations than in the measurements." That is, the text simply states that the simulations replicate the main experimental observations; intermodal coupling leads to the observed differences between the SM and MM spectra.

Corresponding modification in the revised manuscript:

We have revised the text to state that the simulations provide some conceptual support for the mechanisms:

(Line 285) We have revised: *"The suppression of the resonant sidebands that is observed in experiments with the MM laser is also seen in simulations, providing conceptual support for the possible mechanisms underlying the spectral changes, but is not as dramatic (Fig. S12)."*

Comment (8): Overall, the manuscript presents an interesting work, but the evidence does not yet support a broadly significant advance suitable for Nature Communications. Publication would require sharper novelty positioning, quantitative proof of high-even-order dominance, phase-sensitive evidence if coherence is implied, and controlled tests of the proposed multimode mechanisms.

Response (8): We believe that the revised manuscript addresses these points directly:

- Regarding novelty positioning, we make clear that this is the first experimental study of multimode lasers with higher-order dispersion. After thorough characterization of soliton pulses in individual TMs (essentially separate lasers), we generate new pulsed states that self-organize through nonlinear intermodal coupling. They consist of tightly-synchronized pulses in different TMs, which are naturally not identical to the SM solitons. We do not claim that these states are coherent STML states because a coherent spatiotemporal packet does not exist throughout the laser cavity. We also do not provide evidence of phase-locked operation. On the other hand, their emergent nature, tight temporal and spectral synchronization, and short pulse duration indicate that these states may well be a step toward STML with higher-order dispersion. We are confident that there will be significant interest in such states in the MM laser community.
- Quantitative proof of PHEOD dominance is provided in the form of direct estimates of the relevant group delays, along with the strong agreement between measured and predicted spectral sideband positions, which vary dramatically with dispersion order.
- Complete phase coherence of the generated pulses is not claimed.
- Regarding the MM mechanisms, evidence of the proposed pulse-shaping processes comes from i) the experimental observations of spectral, temporal, and spatial changes to the laser state on the transition from SM to MM operation, ii) the measured steady-state behavior and performance of the laser, and iii) the simulation results of Supplementary Note 9. The latter agree reasonably with experimental results and allow partial isolation of effects in the cavity. Of course, more-precise understanding of this complex system will be desirable and will be developed in future work. However, we believe that the main features of the

experiments are accounted for reasonably by the explanations in the paper.

References

R1. Parra-Rivas, P. et al. Pure quartic three-dimensional spatiotemporal Kerr solitons in graded-index media. Phys. Rev. A 109, 033516 (2024).

R2. Liu, Y. et al. Pure-quartic solitons in multimode mode-locked fiber lasers. Opt. Express 33, 30290–30303 (2025).

R3. Liu, Y. et al. Pure high-even-order dispersion solitons in multimode mode-locked fiber lasers. Chaos Solit. Fract. 207, 118014 (2026).

R4. Blanco-Redondo, A. et al. Pure-quartic solitons. *Nat. Commun.* **7**, 10427 (2016).

Our responses to the remaining comments from Reviewer 3 are provided below. Comments from the reviewer are in blue text.

REVIEWERS' COMMENTS

Reviewer #3 (Remarks to the Author):

The authors' response and additional analyses address several technical concerns. In particular, the new spectral-phase, group-delay, dispersion-length, and sensitivity analyses provide reasonable evidence that the applied high-even-order dispersion dominates over the residual β_2 and β_3. The authors have also clearly stated that they do not claim intermodal phase locking, coherent STML, or fully independent per-mode dispersion control. I therefore consider these specific issues adequately clarified.

We are glad these issues are resolved satisfactorily.

Nevertheless, I remain unconvinced that the manuscript establishes a sufficiently broad and mechanistically resolved advance for Nature Communications. The authors correctly identify the novelty as the first experimental realization of synchronized multimode PHEOD states. However, pure-quartic solitons, PHEOD soliton lasers, and solitons governed by arbitrary negative even-order dispersion are already established [1–3]. Multimode synchronization and STML are also established frameworks [4,5], while multimode pure-quartic and PHEOD laser states have already been investigated theoretically [6,7]. The present advance is therefore primarily the experimental combination of these established elements, rather than a new soliton class, synchronization principle, or dispersion-engineering concept.

We would beg to differ with the reviewer regarding how "established" multimode synchronization and STML are. The first STML was demonstrated less than 10 years ago, with the first theoretical treatment six years ago – this is an area still in its scientific infancy. The extension of synchronization and mode-locking to the spatial domain is still much more challenging than the well-established locking of longitudinal modes. Spatiotemporal nonlinear effects are much more difficult to control than effects in lower-dimensional systems.

The field of high even-order solitons is proceeding on a similar timeline and trajectory.

The first theoretical treatments of the combination of these two areas only appeared in the last year. Without any experiments, there is no way to know if the theory is accurate. Thus, this work is particularly timely for the field of nonlinear waves. The experimental realization of a new class of nonlinear waves is always a major challenge, so the demonstration is a significant advance in our opinion. Considering that it extends two major new directions in the field, we expect that there will be considerable interest in

this work.

The authors emphasize that the multimode state self-organizes through nonlinear coupling. This defining interpretation remains insufficiently demonstrated. In the low-energy simulation, Kerr-mediated interactions and saturable-absorber gating are reduced simultaneously, so their individual roles cannot be distinguished. The single-mode/multimode comparison shows that multimode interactions can contribute to spectral asymmetry and sideband suppression, but the simulated suppression is weaker than in experiment. Moreover, this comparison assumes ideal $\beta_2 = \beta_3 = 0$, whereas the experiment uses a common phase mask and retains mode-dependent residual dispersion. These simulations establish plausibility, but not a uniquely identified or quantitatively validated synchronization mechanism.

This distinction is important because the TMDC already provides periodic linear compensation that prevents unbounded modal walk-off and restores temporal overlap once per round trip. The present results do not clearly establish whether the observed state is a distinct nonlinear multimode attractor or a synchronized cavity state primarily maintained by engineered delay compensation and common saturable-absorber gating. Independent numerical removal of XPM, other intermodal nonlinear terms, and saturable-absorber-mediated coupling—using the actual shared-mask residual dispersions—would be needed to support the stronger self-organization interpretation.

We believe that there is clear and strong evidence for the interpretation of synchronized states in terms of self-organization. The spatial, temporal, and spectral profiles of the lasing state all change simultaneously at a threshold pump power (i.e., threshold nonlinear effects in the cavity), and they vary with power above the threshold. Simulations are consistent with experimental results. The precise and quantitative roles of the distinct physical effects that underlie the self-organized behavior are not determined, but it seems clear that nonlinear self-organization occurs.

To further examine the roles of the Kerr nonlinearity and the SA, we performed two additional controlled simulations. First, we removed the intermodal nonlinear terms (XPM and FWM) and the corresponding results are shown in Fig. R1a, b. Although the TMDC still periodically resets the accumulated modal walk-off, a substantial relative delay remains between LP02 and LP01, and the temporal profiles develop pronounced satellite and multi-peak structures. As a result, the system does not recover the stable synchronized state obtained in the full model (Supplementary Fig. 2c). We then removed the SA effect, and the results are shown in Fig. R1c, d. In this case, the relative delays among the modes become markedly more irregular during the round-trip evolution, and the temporal profiles undergo severe broadening and fragmentation, so that no stable localized pulsed state is obtained. These results show that periodic linear compensation alone is insufficient; both intermodal Kerr nonlinearity and the SA contribute to the formation and stabilization of the synchronized state.

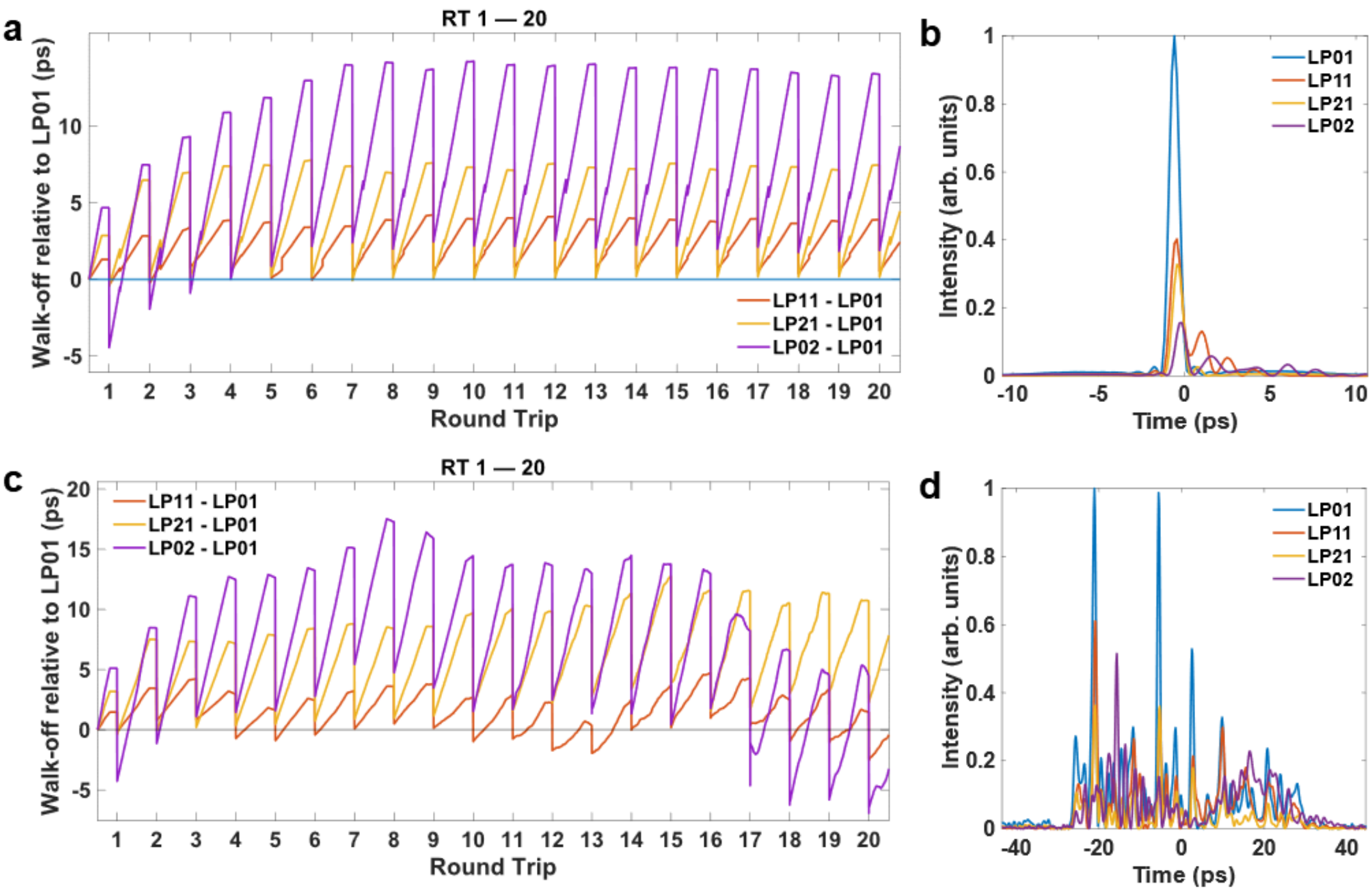

Fig. R1 | Controlled simulations with selected nonlinear and SA effects removed. **a, b,** Intracavity relative modal walk-off over 20 round trips and the corresponding temporal profiles when the intermodal nonlinear terms (XPM and FWM) are removed while SPM is retained. **c, d,** Corresponding intracavity relative modal walk-off and temporal profiles when the SA effect is removed.

With all due respect to the reviewer, he or she previously agreed (first point, above) that $\beta_2 = \beta_3 = 0$ to an excellent approximation, and that independent modal phase control is not claimed. In any case, we don't know why it would be important to demonstrate modal phase control in the present work, which the reviewer suggests is the case. It is scientifically sufficient, and more general, to demonstrate the new synchronized states without the requirement of independent modal phase control.

Corresponding modification in the Supplementary Information:

We have added Supplementary Fig. 4 and the corresponding discussion in Supplementary Note 3 to further clarify the roles of intermodal nonlinear interactions and the saturable absorber in pulse synchronization (Lines 162-178).

I accept the authors' clarification that performance optimization is not the objective, and I do not regard the absence of superior pulse energy or duration as a methodological flaw. However, once practical performance and energy scaling are excluded from the central claims, the impact must rest mainly on the discovery and understanding of a new physical state. Here, the state is explicitly not coherent STML, does not involve independent modal phase control, and its governing nonlinear mechanism remains unresolved.

Overall, the manuscript presents a technically sophisticated experimental implementation of synchronized multimode PHEOD pulse trains. However, the underlying PHEOD physics, multimode synchronization framework, and theoretical multimode PHEOD states are already established, while the present work does not demonstrate coherent STML, a new scaling law, independent modal functionality, or a uniquely resolved synchronization mechanism. I therefore remain unconvinced that the advance reaches the level of general significance expected for Nature Communications, although it would be suitable for a focused journal in ultrafast, nonlinear, or multimode photonics.

We appreciate the acknowledgment that this work presents experimental observation of a new physical state. While the manuscript does not claim observation of coherent STML, we think it is important to point out that there is extensive discussion in the text, presaged in the abstract and introduction, regarding the relationship of the present work to STML. That discussion should make clear that the results are quite "close" to STML. It is even possible that the pulses are coherent, and some researchers would already refer to the observed states as STML. However, we choose to be scientifically conservative in our claims because we cannot prove that STML is occurring. The reviewer does not acknowledge this discussion, which adds to the general significance of the work. We expect this work to inspire follow-up research to determine if the pulses are STML, and to generate discussion in the field regarding what should be considered STML.
